# Uncovering the active constituents and mechanisms of Rujin Jiedu powder for ameliorating LPS-induced acute lung injury using network pharmacology and experimental investigations

**DOI:** 10.3389/fphar.2023.1186699

**Published:** 2023-05-11

**Authors:** Yuhui Ma, Hong Xu, Gang Chen, Wei Liu, Chao Ma, Jialei Meng, Lin Yuan, Xu Hua, Guangbo Ge, Ming Lei

**Affiliations:** ^1^ Department of Critical Care Medicine, Seventh People’s Hospital of Shanghai University of Traditional Chinese Medicine, Shanghai, China; ^2^ Shanghai Frontiers Science Center of TCM Chemical Biology, Institute of Interdisciplinary Integrative Medicine Research, Shanghai University of Traditional Chinese Medicine, Shanghai, China; ^3^ Key Laboratory of Liver and Kidney Diseases (Ministry of Education), Department of Pharmacy, The SATCM Third Grade Laboratory of Traditional Chinese Medicine Preparations, Shanghai Key Laboratory of Traditional Chinese Clinical Medicine, Shuguang Hospital Affiliated to Shanghai University of Traditional Chinese Medicine, Shanghai, China; ^4^ Shanghai TCM-Integrated Hospital, Shanghai University of Traditional Chinese Medicine, Shanghai, China

**Keywords:** Rujin Jiedu powder (RJJD), acute lung injury, anti-inflammatory, apoptosis, network pharmacology

## Abstract

**Background:** Acute lung injury (ALI) is a common clinical disease with high mortality. Rujin Jiedu powder (RJJD) has been clinically utilized for the treatment of ALI in China, but the active constituents in RJJD and its protective mechanisms against ALI are still unclear.

**Methodology:** ALI mice were established by intraperitoneal injection of LPS to test the effectiveness of RJJD in treating ALI. Histopathologic analysis was used to assess the extent of lung injury. An MPO (myeloperoxidase) activity assay was used to evaluate neutrophil infiltration. Network pharmacology was used to explore the potential targets of RJJD against ALI. Immunohistochemistry and TUNEL staining were performed to detect apoptotic cells in lung tissues. RAW264.7 and BEAS-2B cells were used to explore the protective mechanisms of RJJD and its components on ALI *in vitro*. The inflammatory factors (TNF-α, IL-6, IL-1β and IL-18) in serum, BALF and cell supernatant were assayed using ELISA. Western blotting was performed to detect apoptosis-related markers in lung tissues and BEAS-2B cells.

**Results:** RJJD ameliorated pathological injury and neutrophil infiltration in the lungs of ALI mice and decreased the levels of inflammatory factors in serum and BALF. Network pharmacology investigations suggested that RJJD treated ALI via regulating apoptotic signaling pathways, with AKT1 and CASP3 as crucial targets and PI3K-AKT signaling as the main pathway. Meanwhile, baicalein, daidzein, quercetin and luteolin were identified as key constituents in RJJD targeting on the above crucial targets. Experimental investigations showed that RJJD significantly upregulated the expression of p-PI3K, p-Akt and Bcl-2, downregulated the expression of Bax, caspase-3 and caspase-9 in ALI mice, and attenuated lung tissue apoptosis. Four active constituents in RJJD (baicalein, daidzein, quercetin and luteolin) inhibited the secretion of TNF-α and IL-6 in LPS-induced RAW264.7 cells. Among these components, daidzein and luteolin activated the PI3K-AKT pathway and downregulated the expression of apoptosis-related markers induced by LPS in BEAS-2B cells.

**Conclusion:** RJJD alleviates the inflammatory storm and prevents apoptosis in the lungs of ALI mice. The mechanism of RJJD in treating ALI is related to the activation of PI3K-AKT signaling pathway. This study provides a scientific basis for the clinical application of RJJD.

## Introduction

Acute lung injury (ALI) is a clinical critical disease with high morbidity and mortality (Matthay et al., 2019; Jiang et al., 2023). The pathophysiological features of ALI include massive infiltration of inflammatory cells, diffuse interstitial and alveolar edema, and decreased pulmonary compliance, which may induce acute respiratory distress syndrome (ARDS) to some extent (Butt et al., 2016; Wang et al., 2023). ARDS is a severe life-threatening disease with a mortality rate as high as 40% (Thompson et al., 2017; Narota et al., 2023). ALI/ARDS has many common causes, such as severe pulmonary infection, pulmonary contusion, sepsis and severe pancreatitis (Kumar, 2020). Currently, the treatment of ALI/ARDS mainly includes mechanical ventilation and restrictive fluid management, and no specific pharmacological therapy has been established (Devaney et al., 2011; Zhen et al., 2022). Therefore, the development of a safe and effective drug to treat ALI is imperative.

Long-term clinical usage of Chinese herbal medicines (CHMs) has led to the accumulation of valuable experience in the treatment of ALI (Zhang et al., 2021). Rujin Jiedu (RJJD) powder, a classical prescription in ancient China, was first mentioned in Jing Yue Quan Shu, a famous medical monograph of the Ming Dynasty. RJJD was believed to treat lung abscesses by purging fire and detoxification. RJJD is composed of six herbs: *Platycodon grandiflorum* (Jacq.) A. DC. (Jiegeng, JG), *Glycyrrhiza uralensis* Fisch*.* (Gancao, GC), *Coptis chinensis* Franch*.* (Huanglian, HL), *Scutellaria baicalensis* Georgi. (Huangqin, HQ), *Phellodendron chinense* Schneid. (Huangbo, HB), and *Gardenia jasminoides* Ellis. (Zhizi, ZZ). According to the record of the Chinese Pharmacopoeia 2020, these herbs function in clearing heat and detoxification and in eliminating phlegm and preventing asthma. Modern pharmacology studies have revealed that HQ and GC are rich in various flavonoids (Zhao et al., 2019; Yue et al., 2021), while alkaloids are characteristic components of HL and HB (Wang et al., 2019; Si et al., 2021); JG is abundant in triterpenoid saponins (Zhang et al., 2022), and ZZ is distinguished by the presence of iridoid glycosides (Chen et al., 2020). These compounds and extracts possess a wide range of pharmacological activities, such as anti-inflammatory, antibacterial, antiviral, antioxidant, and antitumor activities (Luo et al., 2019). However, the primary effective substances of RJJD against ALI and their regulatory mechanism remain to be explored.

The multiple constituents in RJJD have seriously hindered the deeper understanding of the active substances and the underlying mechanisms of this CHM against ALI. Network pharmacology offers a practical tool for deciphering the multi-component, multi-pathway, and multi-target network synergies between CHM and a particular human disease (He et al., 2022). Network pharmacology has been widely used for concretizing and deciphering the key effective constituents and related molecular mechanisms of Chinese medicines, which strongly facilitates the modern researches of Chinese medicines (Zhao et al., 2023). In this study, UHPLC-Q-Orbitrap HRMS was used to global analysis the chemicals in RJJD, while the potential targets and signaling pathways of these chemicals for the treatment of ALI were predicted by network pharmacology. Gene Ontology (GO) enrichment demonstrated that the regulation of apoptotic signaling in biological processes was closely associated with RJJD against ALI. Kyoto Encyclopedia of Genes and Genomes (KEGG) enrichment analysis revealed that PI3K-AKT signaling pathway was the critical mechanism of RJJD in the treatment of ALI. In addition, AKT1 and CASP3 (Caspase-3) were identified as key targets based on protein-protein interaction (PPI) analysis. Inflammation and infection are considered as the primary reasons to trigger ALI/ARDS, while epithelial cell damages including apoptosis and necrosis are the main features of acute alveolar injury (Albertine et al., 2002). These findings strongly encourage us to investigate further the active constituents and mechanisms by which RJJD ameliorates LPS-induced ALI from anti-inflammatory and anti-apoptotic perspectives.

The aims of this study were to uncover the active constituents and underlying mechanisms of RJJD for ameliorating LPS-induced ALI using both network pharmacology and experimental investigations. For these purposes, LPS-induced ALI mice model was established for evaluating the anti-inflammatory effect of RJJD *in vivo*. Next, a “key compound-key target-pathway” network was constructed using network pharmacology to find the potential active constituents in RJJD and related key signaling pathways for treating ALI. Subsequently, we validated that RJJD could exert anti-apoptotic effects and ultimately protect lung tissue in ALI mice via activating the PI3K-AKT signaling pathway. Finally, the anti-inflammatory and anti-apoptotic effects of the identified key constituents in RJJD were tested at the cellular level. This study evaluated the efficacy of RJJD against ALI both *in vivo* and *in vitro*, and provided a pharmacological basis for its treatment of ALI.

## Materials and methods

### Chemicals and reagents

*S. baicalensis* Georgi (No. 210826), *G. uralensis* Fisch. (No. 210701), *P. grandiflorum* (Jacq.) A. DC. (No. 210601), *C. chinensis* Franch. (No. 210629), *P. chinense* Schneid. (No. 210707) and *G. jasminoides* Ellis (No. 210625) were provided by Shanghai Kangqiao Traditional Chinese Medicine Beverage Co., Ltd. Lipopolysaccharide (LPS, from *Escherichia coli* 0111: B4) and dexamethasone (Dex, D4902) were purchased from Sigma‒Aldrich (St Louis, MO, United States). Quercetin (BP1187-20 mg, purity ≥ 98%), baicalein (BP0232-20 mg, purity ≥ 98%), luteolin (BP0896-20 mg, purity ≥ 98%), and daidzein (BP0445-20 mg, purity ≥ 98%) were purchased from Purifa (Purifa Biotechnology Inc., Chengdu, CHN).

### Preparation of RJJD

The composition of the RJJD formula is shown in Supplementary Table S1. The preparation of RJJD powder is described below. The crude RJJD drug weighed 0.31 kg. The drug was boiled in 2.48 L (8 times, w/v) of pure water for 45 min and the filtrate was obtained. The residue was then boiled in 1.86 L of pure water (6 times, w/v) for 30 min to obtain the filtrate. The two filtrates were combined and freeze-dried in a LABCONCO FreeZone 12 L Freeze Dryer (LABCONCO, Germany) to obtain 0.108 kg dry powder. The extract rate (%) = extract of dry powder/total quality of crude drugs. RJJD powder was extracted at a rate of 34.86%. The specimens (No. 2021110502) were deposited in the central laboratory of the Seventh People’s Hospital of Shanghai University of Traditional Chinese Medicine.

### LPS-induced ALI mouse model

All animal experimental protocols were approved by the Animal Care and Use Committee of Shanghai University of Traditional Chinese Medicine (No. PZSHUTCM220613019). Male C57BL/6J mice (6–8 weeks old, weight: 20 ± 2 g) were purchased from Hangzhou Ziyuan Laboratory Animal Technology Co., Ltd. All animals were raised in a specific pathogen-free (SPF) environment. After 7 days of acclimation, the mice were randomly divided into 5 groups (*n* = 16/group): the Control group (0.9% saline treatment), LPS group (12.5 mg/kg LPS given by intraperitoneal injection), RJJD low-dose group (RJJD 1.9 g/kg/d before LPS intraperitoneal injection), RJJD high-dose group (RJJD 4.75 g/kg/d before LPS intraperitoneal injection) and Dex group (Dex 2 mg/kg/d before LPS intraperitoneal injection). The experimental doses of RJJD in animals were converted from human clinical trial doses. The conversion accounted for body surface area (BSA) scaling; an adult’s body weight was taken to be 70 kg, and the conversion factor was 12.33 (Blanchard and Smoliga, 2015). All drug treatment groups were administered intragastrically once daily for two consecutive days. LPS was injected intraperitoneally within 1 h of the last dose. Six hours after LPS injection, blood samples were collected from the first batch of animals. Twenty-four hours after LPS injection, bronchoalveolar lavage fluid (BALF) and lung tissue were collected from the second batch of animal models.

### Histopathologic analysis

Lung tissues from mice were fixed in 10% neutral-buffered formalin for 48 h. After alcohol gradient dehydration and transparency, the samples were immersed in paraffin. Then, the paraffin blocks were cut to generate 4 μm thick sections, which were stained with hematoxylin and eosin (H&E). The sections were photographed with an optical microscope. Observers assessed the degree of inflammatory cell infiltration, pulmonary edema, alveolar lumen integrity, alveolar wall thickening, and lung tissue congestion according to the following scoring criteria: 0, no injury; 1, <25% injury; 2, 25%–50% injury; 3, 50%–75% injury; and 4, diffuse injury (Zhang et al., 2021).

### Lung wet/dry ratio

The lung wet/dry ratio is the basis for the evaluation of pulmonary edema. When the lungs were isolated, right middle lobe tissue from each group of mice was accurately weighed, and the values were recorded as the wet weight. The lung tissue was placed in an oven at 65°C for 72 h, after which the dried tissue was weighed again. The wet/dry weight ratio (W/D) of the lungs was calculated to evaluate pulmonary edema in each group.

### Collection of bronchoalveolar lavage fluid (BALF)

The mice were sacrificed by injecting an overdose of pentobarbital, and then the neck and trachea fully exposed. BALF was obtained by injecting 0.5 mL PBS from the trachea to the left lung and by repeated flushing of the left lung three times. The samples were centrifuged at 3,000 rpm for 10 min (min) at 4°C and the supernatant was harvested. The cell pellet was resuspended in saline and the cells were counted using a hemocytometer. The total protein concentration of the BALF was determined by a BCA protein assay kit (Thermo Scientific, MA, United States).

### Assay of MPO activity

The lung tissue of mice in each group was accurately weighed, and tissue homogenates were prepared at 4°C. The MPO activity of the homogenates was measured using an MPO activity assay kit according to the manufacturer’s instructions (Nanjing Jiancheng, Nanjing, China).

### Detection of inflammatory factors by ELISA

Blood samples from mice were kept at room temperature for 30 min, after which serum samples were separated by centrifugation at 3,500 rpm for 15 min at 4°C. For *in vitro* experiments, the supernatants of RAW264.7 cells were centrifuged at 4,000 rpm for 10 min and collected for subsequent experiments. The levels of TNF-α, IL-6, IL-1β and IL-18 in the serum, BALF and supernatants were determined using ELISA kits (Abcam, Shanghai, China) according to the manufacturer’s instructions.

### Global chemical profiling of RJJD

The chromatography analysis was performed on a UHPLC-HRMS (ultrahigh-performance liquid chromatography-high resolution mass spectrometer) consisting of a Thermo Scientific Dionex Ultimate 3,000 Series RS pump, Thermo Scientific Dionex Ultimate 3,000 Series TCC-3000RS column compartments, a WPS-3000 autosampler, and Q-Exactive Orbitrap system (Thermo Fisher Scientific Inc., Grand Island, NY, United States). Chromatographic separation was performed on a Waters ACQUITY UPLC BEH C18 column (2.1 mm × 100 mm, 1.7 μm). The autosampler was set at 4°C, and the column temperature was set at 40°C. The mobile phase consisted of methanol (A) and a 0.1% formic acid aqueous solution (B), which was delivered at a flow rate of 0.3 mL/min. The gradient elution program was set as follows: 0–4 min (4% A), 4–10 min (4%–12% A), 10–30 min (12%–70% A), 30–35 min (70% A), 35–38 min (70%–95% A), 38–42 min (95% A), and 42–45 min (4% A). The injection volume was 2 μL. Xcalibur 4.1 software was used for data collection and analysis. The MS acquisition was operated in the negative ion mode with a resolution of 70,000 FWHM. The scanning range was *m/z* 80–1,200.

### Screening of active components and potential targets of RJJD

The active constituents of RJJD obtained by mass spectrometry were used for composition screening and target acquisition with the help of the Traditional Chinese Medicine Systems Pharmacology Database and Analysis Platform (TCMSP) and the Swiss Target Prediction Database (Gfeller et al., 2014; Ru et al., 2014). Using the TCMSP database, we screened compounds according to an oral bioavailability (OB) ≥ 20% and a drug similarity (DL) ≥ 0.10 based on pharmacokinetically relevant information. Using the SwissADME tool in the Swiss Target Prediction Database to screen active compounds not contained in TCMSP and obtain corresponding targets. The filtering principle is that the GI substitution option in the pharmaceutics parameter is “high,” and at least three options in the druglikeness parameter, Lipinski, Ghost, Veber, Egan, and Muage options, are “yes.” For the Swiss Target Prediction Database, the potential active compounds were entered in SMILES format in this database, and “humans” (*Homo sapiens*) was selected as the species to obtain the potential targets of the constituent compounds. All the collected objects were entered into the UniProt database and given an official name (Apweiler et al., 2004).

Potential ALI-related targets were retrieved from the GeneCards and DisGeNET databases using the search term “acute lung injury” (Piñero et al., 2017). The targets with scores ≥ 20.00 in the search results of GeneCards and all targets of the DisGeNET database were selected. After merging the two disease database targets, duplicate values were deleted to find the targets associated with acute lung injury.

For visualization, the above potential active compounds of RJJD and their potential effector targets were entered into Cytoscape software (Version 3.9.0) to plot the “RJJD-compound-potential target” network analysis diagram (Shannon et al., 2003). In this graph, different nodes represent the potential active compounds and effector targets of RJJD, while the edges of the graph indicate the relationship between the two factors.

To construct a protein-protein interaction (PPI) network to explore the important targets of RJJD for ALI treatment, we first obtained the overlapping targets of RJJD and ALI using a Venn diagram (http://bioinformatics.psb.ugent.be/webtools/Venn/). The STRING database was used to create a PPI network (Mering et al., 2003). The protein category was set as *H. sapiens*. The minimum required interaction score was “highest confidence (0.9),” and the PPI network was obtained using the default setting for the other parameters. Free targets were removed, and a PPI analysis was conducted. In the PPI network, the larger the node, the larger the degree value, which indicates a better correlation between the protein and the treatment mechanism. Then, the obtained files were imported into Cytoscape, and the core targets were screened using the cytoHubba plug-in (Chin et al., 2014).

### GO function enrichment and KEGG pathway enrichment analyses

The enrichment analysis method was selected from the R package clusterProfiler (https://www.bioconductor.org, version 3.1.1), and the annotated species was selected as “human” (Yu et al., 2012). Gene Ontology (GO) biological process (BP), molecular function (MF) and cellular component (CC) enrichment analyses were performed in R 4.1.1, and Kyoto Encyclopedia of Genes and Genomes (KEGG) enrichment analyses of potential effector targets were also performed; annotation results for which *p <* 0.05 were selected (Ashburner et al., 2000; Kanehisa et al., 2004).

### Immunohistochemistry

The tissue sections were prepared the same way as mentioned in paragraph “histopathologic analysis.” Lung tissue sections (4 μm) were deparaffinized, rehydrated, and then placed in 0.01 M sodium citrate buffer solution for 15 for antigen retrieval. Subsequently, the sections were incubated in 3% hydrogen peroxide (Shanghai State Pharmaceutical Group, Shanghai, China) for 10 min to block endogenous peroxidase activity. After blocking, the sections were incubated with a primary antibody against caspase-3 (1:200; Proteintech, 19677-1-AP) overnight at 4°C, and then exposed to HRP-labeled goat anti-rabbit IgG secondary antibody for 1 h at 37°C. Immunohistochemical staining was observed and recorded under a light microscope (Nikon) at an original magnification of ×200.

### TUNEL staining

The tissue sections were prepared the same way as mentioned in paragraph “histopathologic analysis.” Lung tissue sections (4 μm) were deparaffinized, rehydrated, incubated in proteinase K for 10 min, and then washed three times in phosphate-buffered saline (PBS; 3 min each time). TUNEL staining was performed according to the instructions of the kit (Roche, 11684817910). Nuclei were counterstained with DAPI (1:500). Apoptotic cells indicated by green fluorescence from fluorescein-dUTP-labeled DNA were observed and recorded by fluorescence microscopy. ImageJ software was used to count the number of TUNEL-positive cells.

### Cell culture and treatment

The mouse macrophage cell line RAW 264.7 and the human normal lung epithelial cell line BEAS-2B were obtained from the Cell Bank of the Chinese Academy of Sciences (Shanghai, China). Cells were cultured in DMEM containing 10% fetal bovine serum (FBS) in a humidified incubator at 37°C, with 5% CO_2_. RAW 264.7 cells were stimulated with 100 ng/mL LPS for 4 h, while BEAS-2B cells were stimulated with 1 μg/mL LPS for 24 h, after which the cells were treated with quercetin (15, 30 μM), daidzein (15, 30 μM), baicalein (15, 30 μM), and luteolin (2.5, 5 μM).

### Cell viability assay

RAW 264.7 and BEAS-2B cells were incubated with different concentrations of compounds for 24 h. A Cell Counting Kit-8 (CCK-8) assay kit was used according to the manufacturer’s instructions (Dojindo, Kumamato, Japan) to determine cell viability.

### Western blotting

Total proteins were extracted from lung tissue and BEAS-2B cells in RIPA lysis buffer containing PMSF and protein phosphatase inhibitors. The total protein concentration of each sample was determined using a BCA protein assay kit (Thermo, MA, United States). The protein homogenates were separated on 10% and 12.5% SDS‒PAGE gels and then transferred to PVDF membranes. After blocking for 1 h at room temperature in 5% (m/v) skim milk, the membranes were incubated overnight at 4°C with the following primary antibodies: anti-PI3K (1:1000, #4257, CST, Boston, United States), anti-p-PI3K (1:1000, AP0854, ABclonal, Cummings Park, United States), anti-AKT (1:1000, #4691, CST, Boston, United States), anti-p-AKT (1:1000, #4060, CST, Boston, United States), anti-mTOR (1:1000, #2983, CST, Boston, United States), anti-mTOR (1:1000, #5536, CST, Boston, United States), anti-BAX (1:1000, #14796, CST, Boston, United States), anti-Bcl-2 (1:1000, #3498, CST, Boston, United States), anti-caspase-3 (1:1000, #9662, CST, Boston, United States), anti-caspase-9 (1:1000, #9504, CST, Boston, United States), and anti-GAPDH (1:1000, #5174, CST, Boston, United States). After rinsing with TBST three times, the membranes were incubated with the secondary antibody at room temperature for 1 h, and were then washed again. Finally, the protein bands were visualized using enhanced chemiluminescence (ECL) reagent (Meilunbio, Dalian, China). The grayscale value of each band was measured by ImageJ software.

### Statistical analysis

All experiments were repeated at least three times. The results were expressed as the mean ± standard deviation (mean ± SD) and determined by one-way ANOVA. *p < 0.05* was considered statistically significant. All statistical data were calculated using GraphPad Prism 8 (GraphPad Software, La Jolla, CA, United States).

## Results

### RJJD alleviates LPS-induced acute lung injury in mice

**FIGURE 1:**
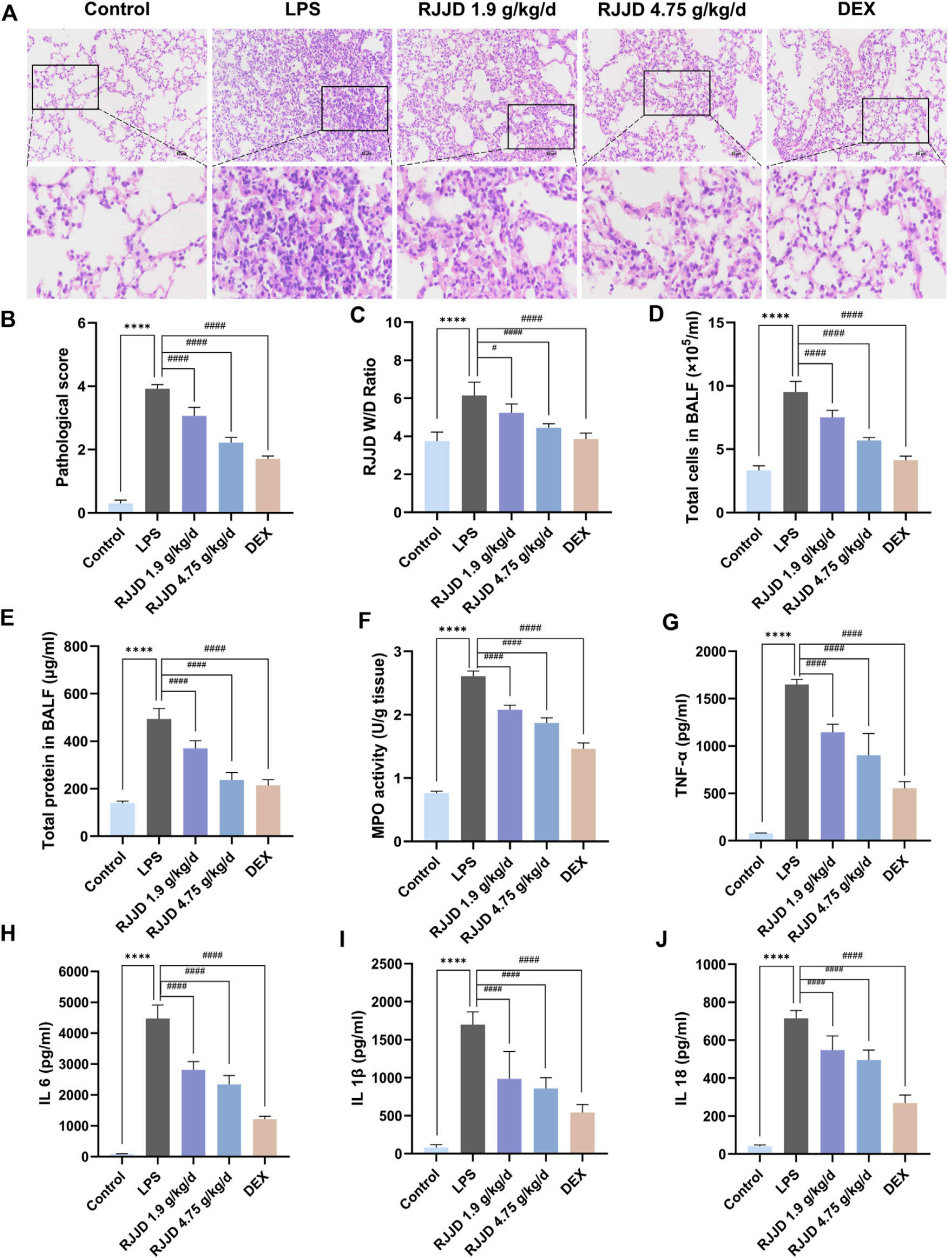
RJJD alleviated LPS-induced ALI mice lung lesions. **(A)** HE staining of lung tissues. Magnification, ×200. **(B)** Lung injury score was determined according to the degree of lung injury in different groups. **(C)** Pulmonary edema was assessed by lung W/D ratio. **(D, E)** The total cell count and total protein concentration of BALF in different groups. **(F)** MPO activity of lung injury in different groups. Serum inflammatory factors **(G)** TNF-α, **(H)** IL-6, **(I)** IL-1β, and **(J)** IL-18 were detected by ELISA. Data was mean ± SD (*n* = 5). ****p < 0.0001 vs. Control group; ^#^ *p <* 0.05 and ^####^ *p <* 0.0001 vs. LPS group.

To verify the protective effects of RJJD on LPS-induced ALI, we first evaluated the pathological changes of lung tissues by H&E staining. Compared with the control group, lung tissue sections from mice treated with LPS showed considerably worse pathological damage, including thickening of the alveolar wall, pulmonary edema, and increased numbers of inflammatory cells. The extent of lung tissue damage and the number of inflammatory cells were both significantly decreased after RJJD (1.9 g/kg/d, 4.75 g/kg/d) and Dex (2 mg/kg/d) treatment (Figure 1A). The ALI pathological scores showed the same trend as the above results. Importantly, high-dose RJJD (4.75 g/kg/d) resulted in a therapeutic efficacy similar to that in the positive control Dex (2 mg/kg) (Figure 1B). Pulmonary edema was assessed according to the lung W/D ratio. The lung W/D ratio in the LPS group was much higher than that in the control group. RJJD (1.9, 4.75 g/kg/d) and Dex (2 mg/kg) treatment significantly reduced the lung W/D ratio (Figure 1C). In addition, inflammatory indicators in BALF are also worthy of attention. The total protein concentration and total cell count in BALF from mice in the LPS group were increased significantly but were decreased in the RJJD (1.9, 4.75 g/kg/d) and Dex (2 mg/kg) treatment groups (Figures 1D, E). MPO is also a marker of inflammation, as it represents the degree of neutrophil infiltration (Cao et al., 2020). A significant increase in MPO activity was observed in the LPS group, and this increase was significantly reduced after RJJD (1.9, 4.75 g/kg/d) and Dex (2 mg/kg) treatment (Figure 1F).

### RJJD decreases the release of serum and BALF inflammatory factors in LPS-induced ALI mice

To evaluate the anti-inflammatory effect of RJJD, we performed ELISA to detect the levels of pro-inflammatory cytokines, including TNF-α, IL-6, IL-1β and IL-18, in the serum. We also detected the contents of TNF-α and IL-6 in BALF. The levels of TNF-α, IL-6, IL-1β and IL-18 in the serum of ALI mice were significantly higher than those in the control group. In contrast, the expression of these cytokines was inhibited by RJJD (1.9, 4.75 g/kg/d) and Dex (2 mg/kg) in a dose-dependent manner (Figures 1G–J). We observed a similar trend in BALF, with elevated levels of TNF-α and IL-6 in the BALF of ALI mice compared to controls. With RJJD dosing, the levels of TNF-α and IL-6 in the BALF of ALI mice were significantly reduced (Supplementary Figure S1).

### Global chemical analysis of RJJD

The chemical constituents in RJJD were globally characterized by UHPLC-Q-Exactive Orbitrap HRMS (Supplementary Figure S2). In brief, 206 constituents in RJJD were identified by comparison with the retention times and MS/MS spectra of the reference standards, related literature, Chemical Book and other databases. The details of each identified constituent in RJJD has been listed in Supplementary Table S2.

### Active components and potential targets of RJJD

Next, we performed an ADME (absorption, distribution, metabolism, and excretion) screen of the 206 constituents in RJJD identified by UHPLC-Q-Exactive Orbitrap HRMS. Fifty-one active compounds of RJJD were obtained after ADME screening. In all, 306 potential targets of RJJD were obtained from the TCMSP and the Swiss Target Prediction databases. Some compounds were shared by two or more herbs. For example, quercetin was shared by Huanglian, Gancao, Huangbo, Huangqin and Zhizi. Details of these components and their corresponding targets are shown in Supplementary Table S3.

### Determination of ALI-related targets

Then, the potential ALI-related targets were retrieved from GeneCards and DisGeNET databases. We retrieved 479 and 93 ALI-related disease targets from GeneCards and DisGeNET, respectively. In all, 521 targets were obtained by deleting duplicate values after merging. Detailed information on these targets is shown in Supplementary Table S4.

### Network-based analysis of the effects and mechanisms of RJJD

**FIGURE 2:**
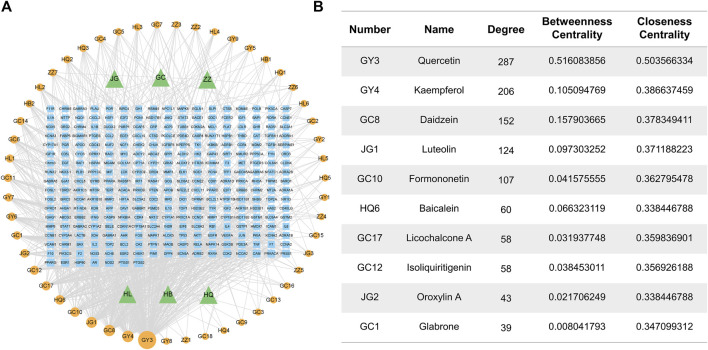
Herb-compound-target network of RJJD. **(A)** The RJJD-compound-target network. The green triangles represent the herb; the orange squares represent the potential targets; the blue circulars represent the drug ingredients. **(B)** Top 10 compounds in terms of degree value.

To identify the most important active compounds in RJJD, we constructed an “RJJD-compound-target” network (Figure 2A). This network consists of 353 nodes (6 herbs, 51 active compounds, 296 targets), connected by 2,612 interactions. The top 10 active compounds (quercetin, kaempferol, daidzein, luteolin, formononetin, baicalein, licochalcone A, isoliquiritigenin, oroxylin A and glabrone) were selected according to degree values, betweenness centrality and closeness centrality (Figure 2B).

**FIGURE 3:**
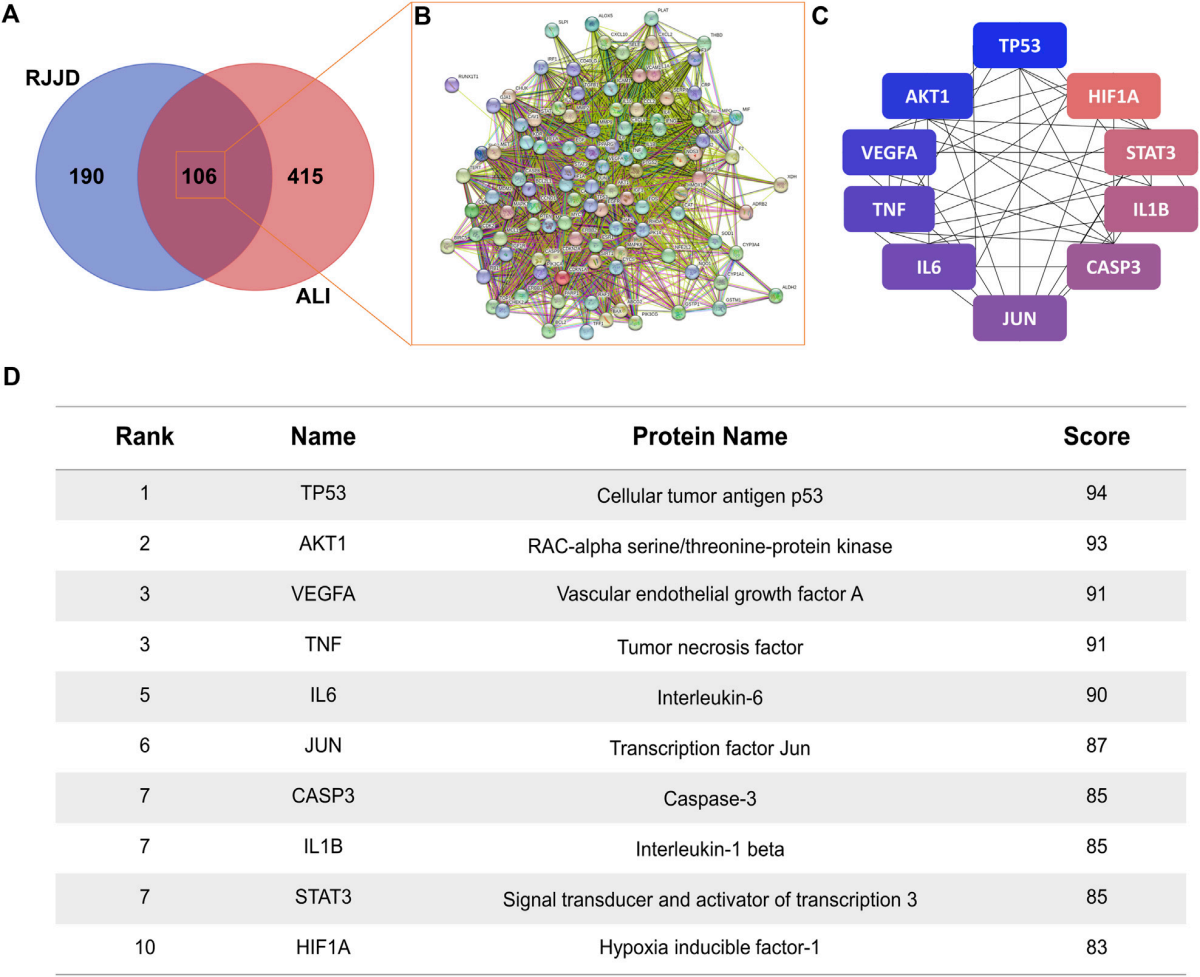
Protein-protein network analysis. **(A)** The Venn diagram of RJJD (296 genes) and ALI (521 genes) with 106 overlapping genes. **(B)** PPI network of the 106 overlapping genes. **(C)** The top ten targets of RJJD in the treatment of ALI. The greater the degree value, the bluer the color. **(D)** Top 10 targets in terms of degree value.

The screened RJJD active components were crossed with the targets of ALI, and 106 common targets were obtained (Figure 3A). The common targets were imported into the STRING 11.0 platform to build a PPI network (Figure 3B). The PPI network contained 106 nodes and 2,655 edges, and the average node degree was 50.1, with a PPI enrichment *p*-value *<* 1.0e-16. The Cytoscape plugin cytoHubba was used to rank nodes in a network by their network features. The top ten targets were TP53, AKT1, VEGFA, TNF, IL6, JUN, CASP3, IL1B, STAT3 and HIF1A, which indicates their significance in the network (Figures 3C, D).

**FIGURE 4:**
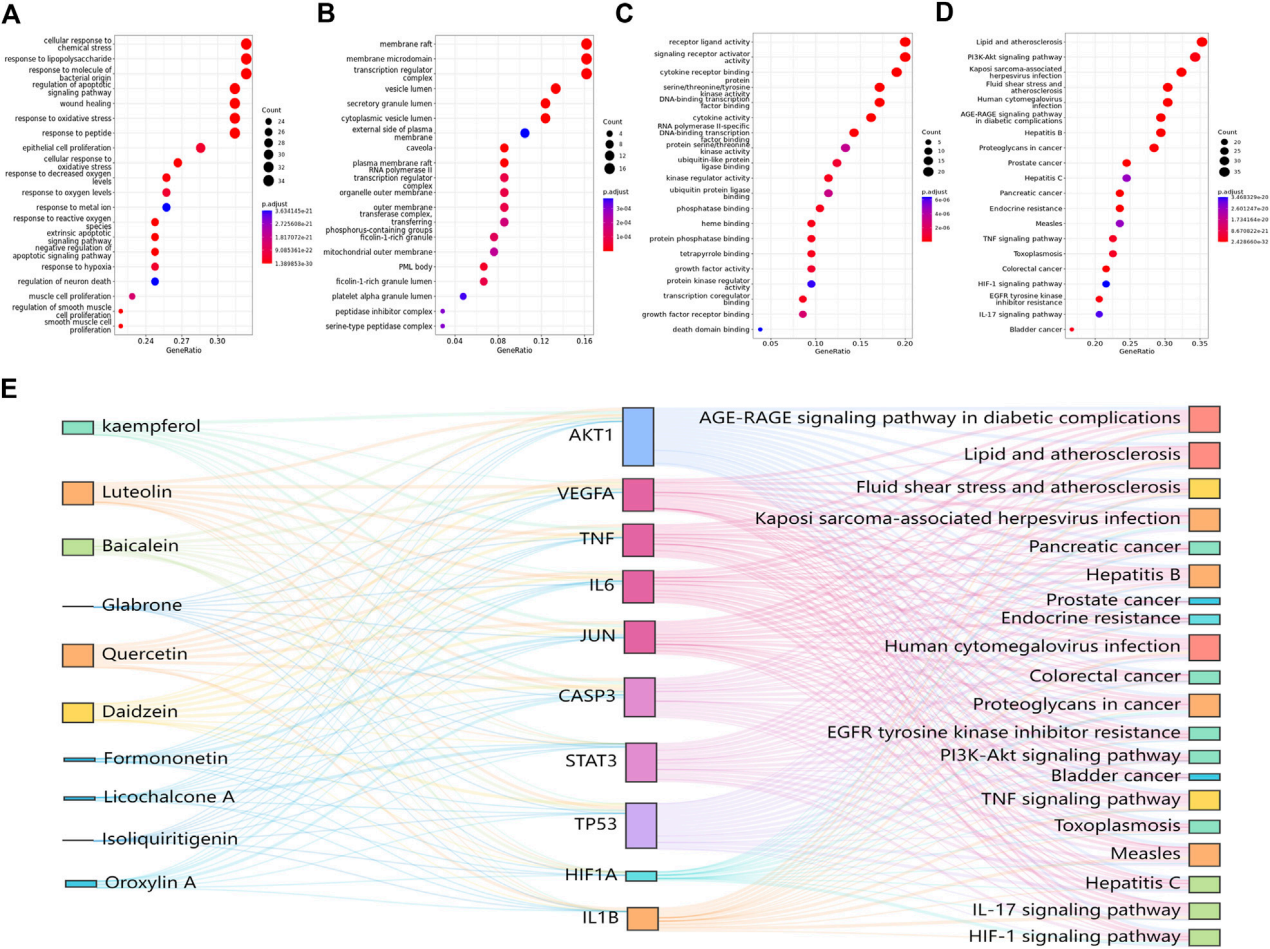
GO and KEGG pathway enrichment analysis results. **(A)** BP enrichment analysis. **(B)** MF enrichment analysis. **(C)** CC enrichment analysis. **(D)** KEGG pathway enrichment analysis. **(E)** The Sankey diagram reveals the relationship between compounds, targets, and pathways. The left blocks represent the ingredients, the middle blocks represent the targets, and the right blocks represent the pathways.

GO functional enrichment analysis and KEGG pathway analysis of key targets were performed. All terms were screened at *p <* 0.05. The top 20 items and pathways with significant *p* values were converted into a bubble chart (Figures 4A–D). As shown in Figure 4A, the top 20 biological process terms included the response to lipopolysaccharide and the regulation of apoptotic signaling pathway, which suggests that RJJD may play an important role in the treatment of ALI by modulating these biological processes. In addition, 146 pathways were identified by KEGG analysis (Figure 4D). Pathways such as the PI3K-AKT signaling pathway, IL-17 signaling pathway and TNF signaling pathway were mostly related to ALI.

Based on the key targets and compounds, we established a “key compound-key target-pathway” network, which comprised interactions among 10 compounds, 10 key targets, and 20 pathways (Figure 4E). In this network, we identified AKT1, CASP3 and TP53 as the three crucial targets for treating ALI, suggesting that these proteins may play an important roles in the treatment of ALI by RJJD.

### RJJD ameliorates apoptosis in LPS-induced ALI mice

**FIGURE 5:**
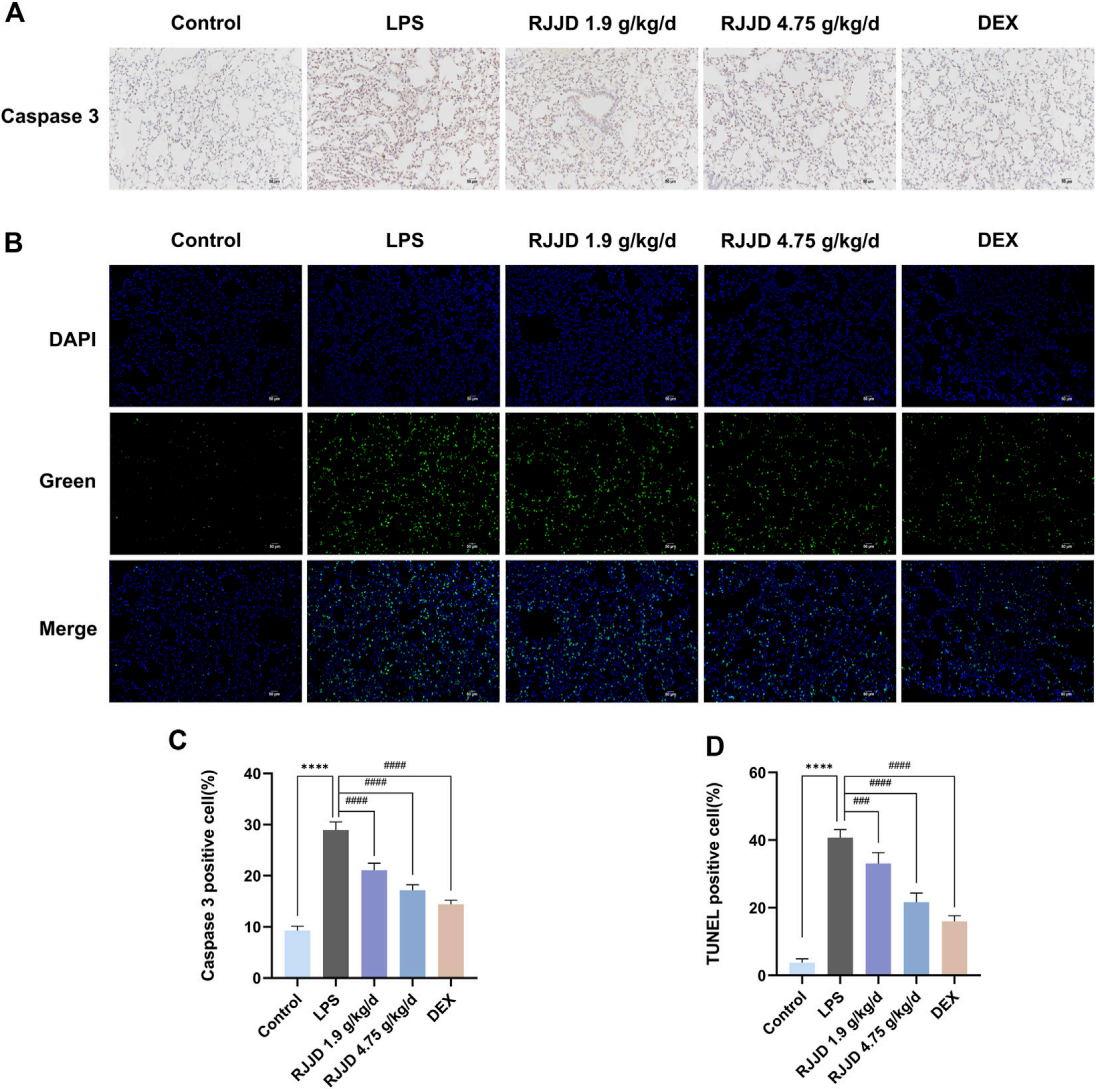
RJJD attenuated LPS-induced ALI mice lung apoptosis. **(A)** Immunohistochemistry was applied to analyze the expression of caspase-3 in lung tissues of different groups of mice. Magnification, ×200. **(B)** TUNEL staining was applied to analyze lung tissue apoptosis. TUNEL-positive cells with green fluorescent nuclei suggested apoptotic cells. Magnification, ×200. **(C)** Statistical results on the percentage of caspase-3 positive cells in lung sections from different groups of mice. **(D)** Statistical results on the percentage of TUNEL-positive cells in lung sections from different groups of mice. Data was mean ± SD (*n* = 5). ****p < 0.0001 vs. Control group; ^###^ *p <* 0.001 and ^####^ *p <* 0.0001 vs. LPS group.

Network pharmacology analysis showed that TP53 and CASP3 were the key targets of RJJD in the treatment of acute lung injury (Figures 3C, D). Therefore, we focused on the anti-apoptotic effect of RJJD to elucidate its protective mechanism in ALI. Immunohistochemistry revealed showed that the number of caspase-3 positive cells in the lung tissue of ALI mice was increased significantly. In contrast, caspase-3 positive cells were decreased considerably after RJJD (1.9, 4.75 g/kg/d) and DEX (2 mg/kg) treatment (Figures 5A, C). Furthermore, TUNEL staining was used to detect apoptosis in lung tissue. A significant increase in apoptotic cells was observed in ALI mice, whereas apoptosis was inhibited in the RJJD (1.9, 4.75 g/kg/d) and Dex (2 mg/kg) treatment groups (Figures 5B, D). These results indicated that RJJD could alleviate lung tissue apoptosis in LPS-induced ALI mice.

### RJJD activates the PI3K-AKT pathway and downregulates CASP3-mediated apoptosis in LPS-induced ALI mice

**FIGURE 6:**
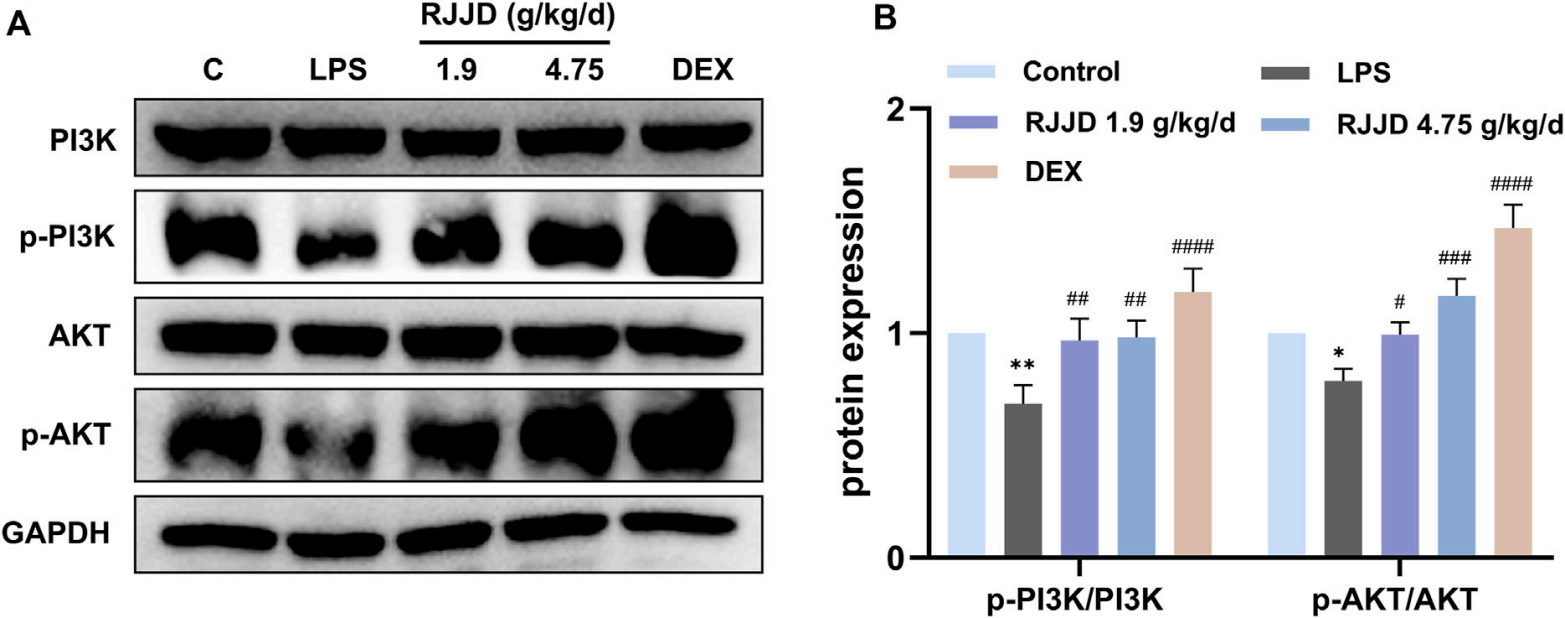
Effect of RJJD on PI3K-AKT signaling pathway in lung tissues. **(A)** Representative Western blot images of PI3K, p-PI3K, AKT and p-AKT. **(B)** The gray values of protein bands were quantified by ImageJ software. Data was mean ± SD (*n* = 3). *p < 0.05 and **p < 0.01 vs. Control group; ^#^ *p <* 0.05, ^##^ *p <* 0.01, ^###^ *p <* 0.001 and ^####^ *p <* 0.0001 vs. LPS group.

**FIGURE 7:**
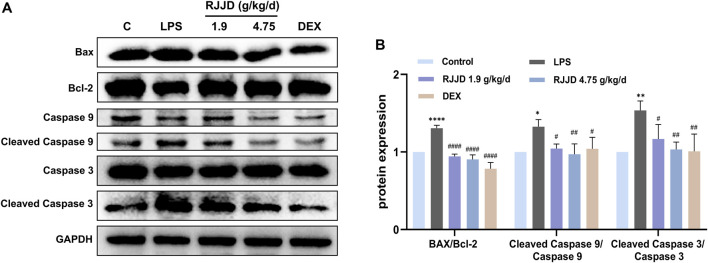
Effect of RJJD on endogenous apoptosis of lung tissues. **(A)** Representative Western blot images of Bax, Bcl-2, caspase-3 and caspase-9. **(B)** The gray values of protein bands were quantified by ImageJ software. Data was mean ± SD (*n* = 3). *p < 0.05, **p < 0.01 and ****p < 0.0001 vs. Control group; ^#^ *p <* 0.05, ^##^ *p <* 0.01 and ^###^ *p <* 0.001 vs. LPS group.

Network pharmacology analysis also suggested that RJJD might inhibit endogenous apoptosis by activating the PI3K-AKT signaling pathway, which in turn, alleviating ALI (Figure 4D). Therefore, we investigated the relationship between RJJD and the PI3K-AKT signaling pathway as well as endogenous apoptosis. Western blotting showed that LPS downregulated PI3K and AKT protein phosphorylation levels in mouse lung tissues. In contrast, RJJD significantly upregulated the phosphorylation levels of PI3K and AKT proteins in a dose-dependent manner (Figures 6A, B). The PI3K-AKT signaling pathway continued to exert anti-apoptotic effects after activation. Next, we determined the expression of apoptosis-related proteins (Bcl-2, Bax, caspase-3 and caspase-9) in mouse lungs by Western blotting. The results showed that LPS upregulated the Bax/Bcl-2 ratio and promoted the expression of caspase-3 and caspase-9 in mouse lung tissues. However, these effects were weakened by RJJD (1.9, 4.75 g/kg/d) and DEX (2 mg/kg) (Figures 7A, B). These data indicated that RJJD activated the PI3K-AKT signaling pathway in the lungs of ALI mice, inhibited endogenous apoptosis and ameliorated lung injury.

### *In vitro* validation of anti-inflammatory and anti-apoptotic effects of key constituents in RJJD

**FIGURE 8:**
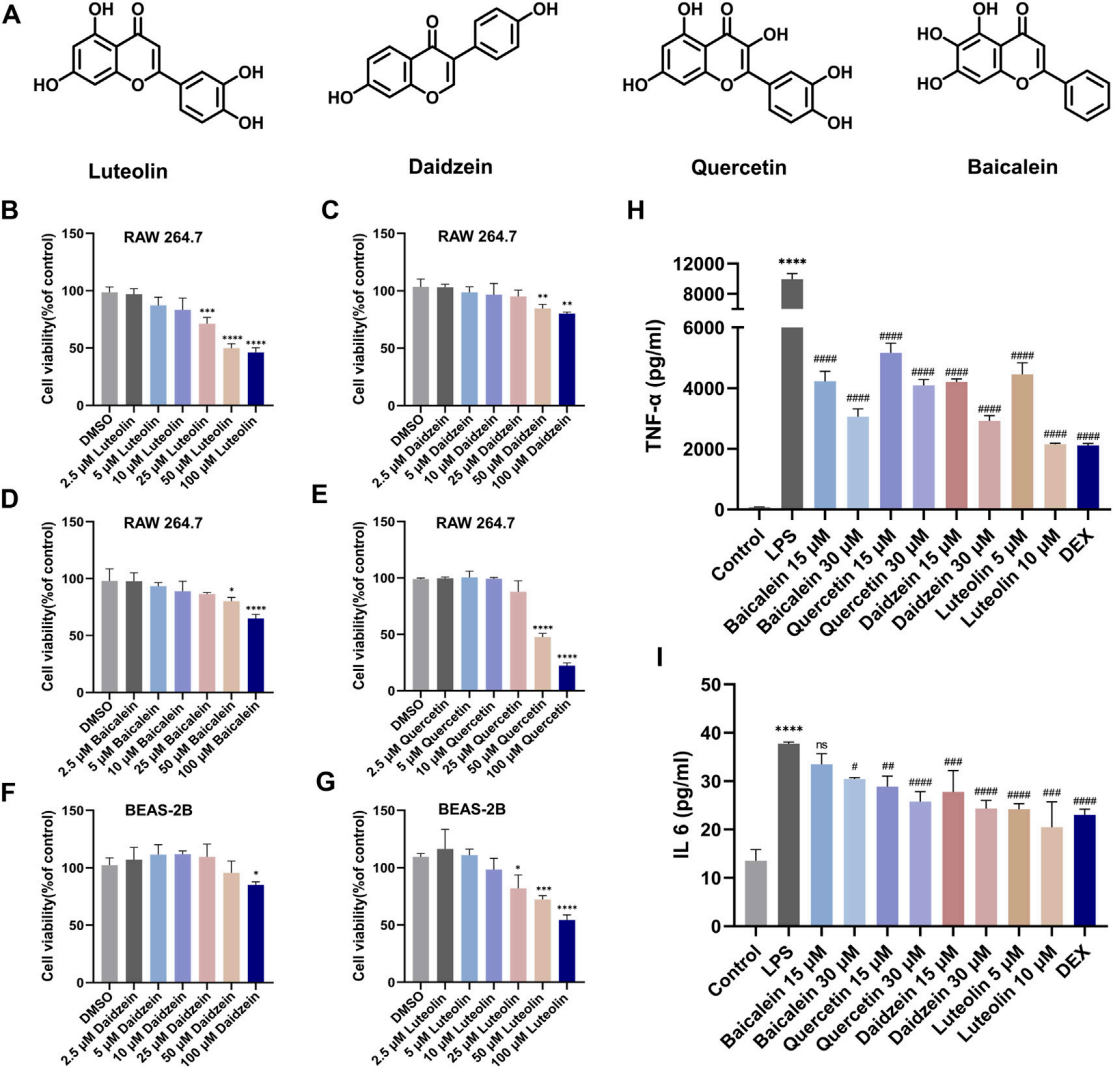
Luteolin, daidzein, quercetin, and baiclein inhibited the secretion of TNF-α and IL-6 from RAW264.7 cells. **(A)** The chemical structure of luteolin, daidzein, quercetin, and baiclein. **(B–G)** RAW264.7 cells were treated with luteolin, daidzein, quercetin, and baiclein for 24 h. BEAS-2B cells were treated with luteolin and daidzein for 24 h. The cytotoxicity of the above compounds to RAW264.7 and BEAS-2B was determined by CCK-8 assay. The levels of **(H)** TNF-α and **(I)** IL-6 in the supernatant of RAW264.7 cells were detected by ELISA. Data was mean ± SD (*n* = 3). *p < 0.05, ***p < 0.001 and ****p < 0.0001 vs. DMSO group and Control group; ^#^ *p <* 0.05, ^##^ *p <* 0.01, ^###^ *p <* 0.001 and ^####^ *p <* 0.0001 vs. LPS group.

**FIGURE 9:**
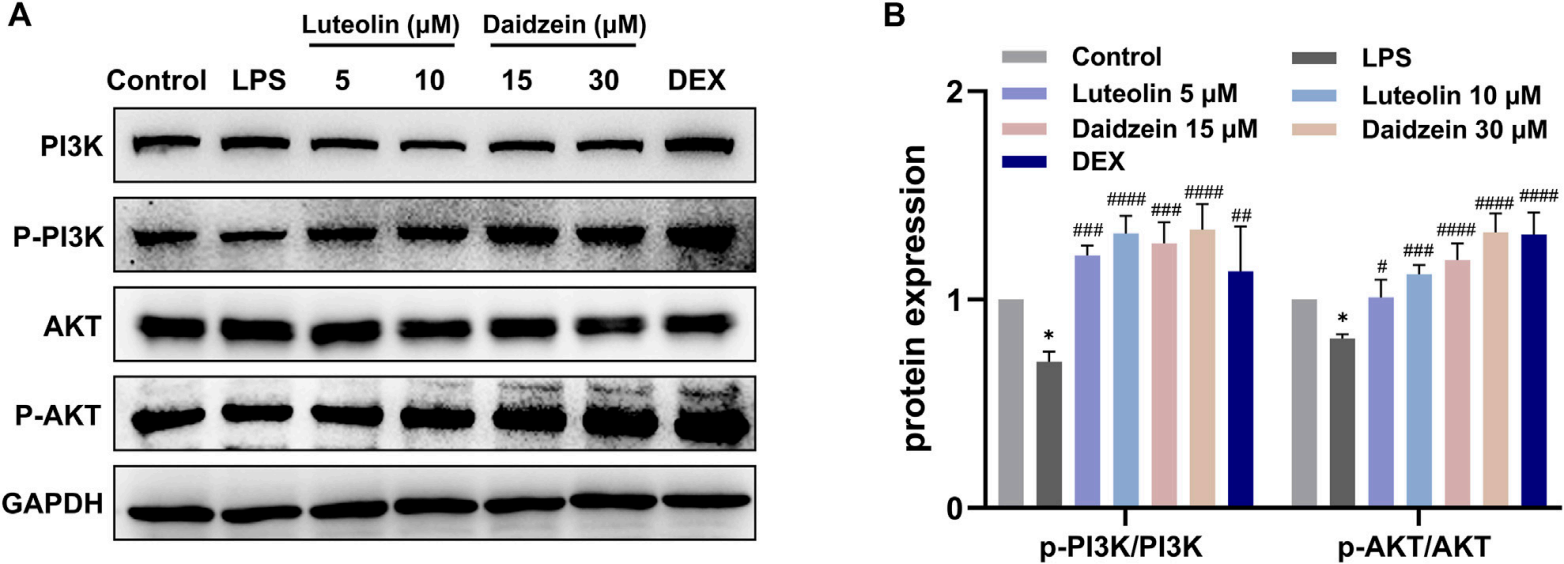
Effect of luteolin and daidzein on PI3K-AKT signaling pathway in BEAS-2B cells. **(A)** Representative Western blot images of PI3K, p-PI3K, AKT and p-AKT. **(B)** The gray values of protein bands were quantified by Image J software. Data was mean ± SD (*n* = 3). *p < 0.05 vs. Control group; ^#^ *p <* 0.05, ^##^ *p <* 0.01, ^###^ *p <* 0.001 and ^####^ *p <* 0.0001 vs. LPS group.

**FIGURE 10:**
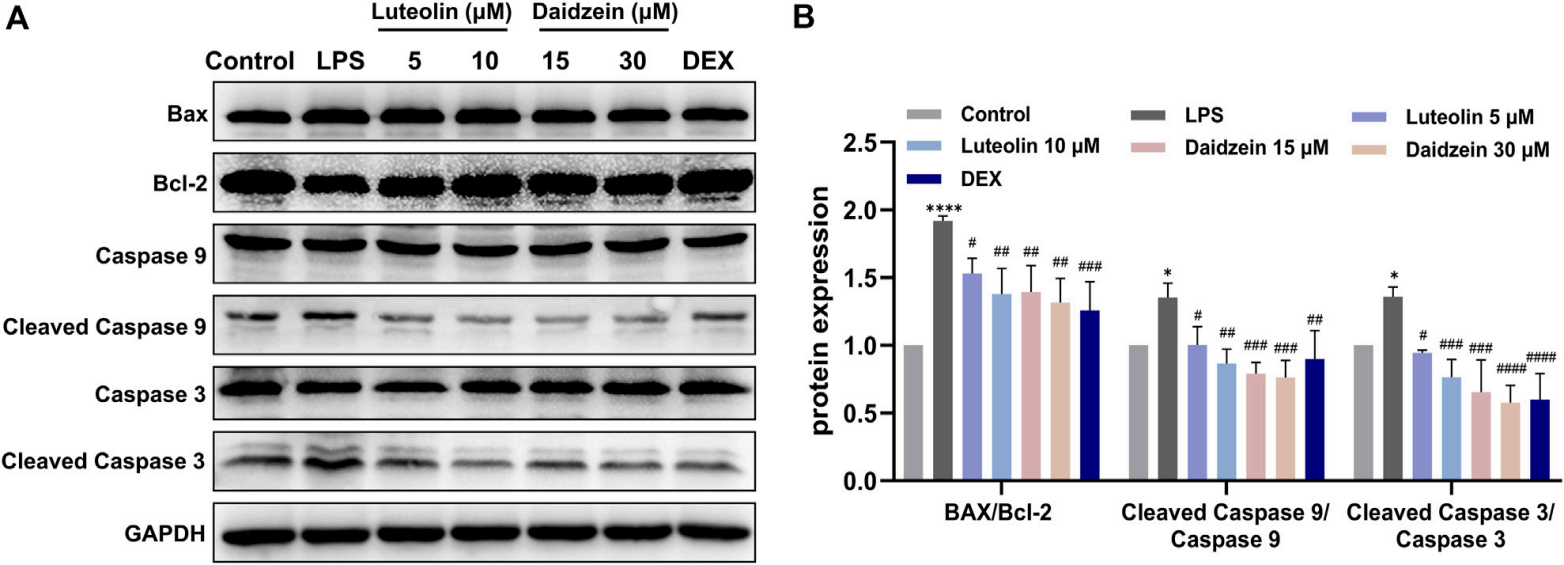
Effect of luteolin and daidzein on endogenous apoptosis in BEAS-2B cells. **(A)** Representative Western blot images of Bax, Bcl-2, caspase-3 and caspase-9. **(B)** The gray values of protein bands were quantified by Image J software. Data was mean ± SD (*n* = 3). *p < 0.05, ****p < 0.0001 vs. Control group; ^#^ *p <* 0.05, ^##^ *p <* 0.01, ^###^ *p <* 0.001 and ^####^ *p <* 0.0001 vs. LPS group.

Next, we evaluated the anti-inflammatory and anti-apoptotic effects of RJJD core active constituents *in vitro*. The anti-inflammatory effects of four key constituents (quercetin, daidzein, luteolin, and baicalein) in RJJD were then investigated in LPS-induced RAW 264.7 cells (Figure 8A). The experimental treatment concentrations of quercetin (15, 30 μM), daidzein (15, 30 μM), baicalein (15, 30 μM), and luteolin (2.5, 5 μM) were determined by CCK-8 (Figures 8B–G). ELISA showed that these four compounds reduced the secretion of pro-inflammatory cytokines (TNF-α and IL-6) in LPS-induced RAW264.7 cells in a dose-dependent manner (Figures 8H, I). Among them, daidzein and luteolin exhibited better anti-inflammatory effects. We also examined whether daidzein and luteolin could inhibit endogenous apoptosis of BEAS-2B cells by activating the PI3K-AKT signaling pathway. The results clearly showed that both daidzein and luteolin upregulated the phosphorylation of PI3K and AKT protein in LPS-induced BEAS-2B cells (Figures 9A, B), downregulated the Bax/Bcl-2 ratio and inhibited the expression of cleaved-caspase-9 and cleaved-caspase-3 (Figures 10A, B). These observations suggested that daidzein and luteolin may act as key anti-inflammatory constituents in RJJD, protecting against ALI via down-regulating the expression of pro-inflammatory cytokines (TNF-α and IL-6) and inhibiting endogenous apoptosis.

## Discussion

Rujin Jiedu powder was one of the classical prescriptions in ancient China and has often been used to treat pneumonia and acute upper respiratory tract infections in recent years. We identified 206 compounds in RJJD, including flavonoids, triterpenoid saponins, alkaloids and amino acids. According to the previously reported literature, many compounds in RJJD have anti-inflammatory effects or the protective effects on organ injury (Hu et al., 2017; Zhu et al., 2021; Liu et al., 2022). However, the therapeutic effect and mechanism of RJJD against ALI are still unclear. In this study, we found that LPS-induced ALI mice exhibited massive infiltration of inflammatory cells, diffuse interstitial and alveolar edema, and decreased pulmonary compliance. However, low- and high-dose RJJD significantly reversed these pathological changes in the lungs of ALI mice.

The main contributing element to ALI, which leads to severe inflammatory damage to lung tissues, is thought to be ongoing lung exposure to bacteria or viruses (Kumar, 2020). Patients with severe ALI often have ARDS, which can progress to acute respiratory failure and multiorgan failure, with a high mortality rate (Confalonieri et al., 2017). ALI/ARDS continues to pose a significant threat to human health worldwide (Bernard et al., 1994). While the pathogenesis of ALI is quite complex, diffuse alveolar damage (DAD) is the main histologic feature of ALI (Ding et al., 2020; Bian et al., 2021). Uncontrolled inflammatory responses cause severe damage to alveolar epithelial cells and pulmonary capillary endothelial cells (Liu et al., 2020). Alveolar integrity is then disrupted, and alveolar-capillary permeability to fluid and proteins is increased (Jiang et al., 2021). The accumulation of protein-rich inflammatory edematous fluid in the alveolar cavity results in pulmonary edema (Gupta et al., 2020). Simultaneously, neutrophils are recruited into the alveolar cavity. Neutrophils and other infected cells secrete high levels of inflammatory factors (TNF-α, IL-6 and IL-8), which in turn induce neutrophil chemotaxis and thus amplify the inflammatory effect (Scozzi et al., 2022). Therefore, effective control of lung inflammation, including neutrophil infiltration and inflammatory mediator release, appears to be a key target for ALI treatment (Grommes and Soehnlein, 2011). In our current study, RJJD significantly decreased neutrophil infiltration in the lungs of ALI mice and showed excellent anti-inflammatory effects. We confirmed the effectiveness of RJJD on LPS-induced ALI mice, but the active constituents and mechanisms require further study.

Network pharmacology was further used to determine the effect and underlying mechanism of RJJD on ALI. Our results demonstrated that the regulation of apoptotic signaling was a key effect of RJJD against ALI and may be accomplished by modulating the PI3K-Akt pathway. The PI3K-AKT pathway is a critical signaling pathway that regulates endogenous apoptosis (Koundouros and Poulogiannis, 2018). In mammalian cells, activation of the PI3K-AKT pathway results in the inhibition of apoptosis and the slowing of secondary cellular damage (Fruman et al., 2017). PI3K-AKT signaling is highly complex. First, receptor tyrosine kinases (RTKs) or G protein-coupled receptors (GPCRs) act as upstream molecules to activate class I PI3Ks. Then, the phosphorylation of PtdIns-4,5-P2 (PIP2) by class I PI3Ks results in the generation of PtdIns-3,4,5-P3 (PIP3). PIP3 recruits AKT to the plasma membrane, which prompts AKT to expose its phosphorylation sites and become activated (Jafari et al., 2019). Similarly, activated AKT phosphorylates Bax, deprives Bax of its pro-apoptotic effect, and prevents the release of the apoptosis-inducing factor cytochrome C from mitochondria, which inhibits caspase-3 and caspase-9 expression and endogenous apoptosis (He et al., 2021). CHM has many active components used to treat ALI that function by modulating the PI3K-AKT pathway and endogenous apoptosis (Hsieh et al., 2018; Wen et al., 2020).

Our findings showed that RJJD could effectively alleviate lung pathological injury in ALI mice, via decreasing serum inflammatory factor levels and inhibiting lung MPO activity. Subsequently, we confirmed four core compounds of RJJD (baicalein, daidzein, quercetin and luteolin) by network pharmacology. In our *in vitro* experiments, the above four compounds significantly inhibited the secretion of TNF-α and IL-6 in the supernatant of LPS-induced RAW264.7 cells. Among them, daidzein and luteolin showed more potent inhibitory effects than the other compounds. Also, we found that in LPS-induced BEAS-2B cells, daidzein and luteolin could activate the PI3K-AKT pathway, suppress the production of pro-apoptotic proteins (Bax, caspase-9 and caspase-3), and increase the expression of anti-apoptotic protein Bcl-2, thereby inhibiting apoptosis. Both experimental results and network pharmacology predictions confirmed that daidzein and luteolin may inhibit PI3K-AKT signaling pathway-mediated endogenous apoptosis, which suggests that RJJD has a pharmacological basis for the treatment of LPS-induced ALI. Our current study still has some shortcomings. For example, the anti-inflammatory and anti-apoptotic mechanisms of RJJD and its active constituents should be investigated more deeply. Furthermore, the anti-inflammatory and anti-apoptotic effects of the active constituents from RJJD should be tested in other immune cells, epithelial cells and endothelial cells. In the future, we will comprehensively analyse the absorbed constituents of RJJD into blood following oral administration, and test the curative effects of the absorbed constituents for fighting ALI.

## Conclusion

In summary, this study uncovered the core active components of RJJD and its potential therapeutic targets on ALI via integrating network pharmacology and experimental pharmacology. RJJD could ameliorate the typical pathological changes of ALI and reduce the serum levels of inflammatory factors *in vivo*. The network pharmacology results suggested that the regulation of apoptotic signaling was the key biological process of RJJD against ALI. Moreover, baicalein, daidzein, quercetin and luteolin were predicted as the key constituents in RJJD against ALI. Finally, the anti-apoptotic effects of RJJD and its key constituents were tested both *in vivo* and *in vitro*, which validated the protective effect of RJJD for treating ALI. Collectively, our results provided clear evidence for supporting the therapeutic effect of RJJD and offered a pharmacological basis for its use in the treatment of ALI, which would very helpful for the modern research and clinical applications of this classic Chinese herbal medicine.

## Figures and Tables

**Table:** 

**ADME**	absorption, distribution, metabolism, and excretion
**ALI**	acute lung injury
**ARDS**	acute respiratory distress syndrome
**BALF**	bronchoalveolar lavage fluid
**BSA**	body surface area
**CHM**	Chinese herbal medicine
**DAD**	diffuse alveolar damage
**Dex**	dexamethasone
**DL**	drug-likeness
**DMEM**	Dulbecco’s Modified Eagle’s Medium
**ECL**	enhanced chemiluminescence
**FBS**	fetal bovine serum
**GO**	gene ontology
**HPLC**	high-performance liquid chromatography
**IL-18**	interleukin-18
**IL-1β**	interleukin-1β
**IL-6**	interleukin-6
**KEGG**	Kyoto Encyclopedia of Genes and Genomes
**LPS**	lipopolysaccharide
**M1**	classically activated macrophages
**M2**	alternatively activated macrophages
**OB**	oral bioavailability
**PBS**	phosphate-buffered saline
**PI3K**	phosphatidylinositol 3-kinase
**PIP2**	PtdIns-3,4-P2
**PIP3**	PtdIns-3,4,5-P3
**PPI**	protein‒protein interaction
**PVDF**	polyvinylidene difluoride membrane
**RJJD**	Rujin Jiedu Powder
**TBST**	tris-buffered saline Tween-20
**TCMSP**	traditional Chinese medicine systems pharmacology database and analysis platform
**TNF-α**	tumor necrosis factor-α
**JG**	*Platycodon grandiflorum* (Jacq.) A. DC.
**GC**	*Glycyrrhiza uralensis* Fisch.
**HL**	*Coptis chinensis* Franch.
**HQ**	*Scutellaria baicalensis* Georgi.
**HB**	*Phellodendron chinense* Schneid.
**ZZ**	*Gardenia jasminoides* Ellis.

## Data Availability

The original contributions presented in the study are included in the article/Supplementary Material, further inquiries can be directed to the corresponding authors.
