# Supplementary material for: Uncovering the active constituents and mechanisms of Rujin Jiedu powder for ameliorating LPS-induced acute lung injury using network pharmacology and experimental investigations
Source: Front Pharmacol. 2023 May 11;14:1186699. doi: 10.3389/fphar.2023.1186699 (PMC10210165; doi:10.3389/fphar.2023.1186699)
Supplement: Supplementary file 1 [file DataSheet4.docx]

Table S4 | ALI-related Targets

| **GeneCards** | **DisGeNET** | **ALL** |
| --- | --- | --- |
| EGFR | ICAM1 | ICAM1 |
| TP53 | PPARG | PPARG |
| KRAS | CXCL2 | CXCL2 |
| TERT | VIP | VIP |
| IL6 | IL17A | IL17A |
| FLT3 | TGFB1 | TGFB1 |
| PTEN | FASLG | FASLG |
| TNF | FAS | FAS |
| MIR126 | TXN | TXN |
| CEBPA | TNFAIP6 | TNFAIP6 |
| KIT | CAT | CAT |
| AKT1 | TFF2 | TFF2 |
| BRAF | NFE2L2 | NFE2L2 |
| STAT3 | ASCL1 | ASCL1 |
| PIK3CA | IL1RL1 | IL1RL1 |
| SFTPC | SFTPD | SFTPD |
| NPM1 | ACVR1 | ACVR1 |
| RUNX1 | EDN1 | EDN1 |
| CDKN2A | MYLK | MYLK |
| ERBB2 | EPHX1 | EPHX1 |
| MIR21 | TFF1 | TFF1 |
| NRAS | APOA1 | APOA1 |
| IL10 | IL1B | IL1B |
| ALK | PLAU | PLAU |
| JAK2 | TNF | TNF |
| CASP8 | ELANE | ELANE |
| RANBP2 | SERPINE1 | SERPINE1 |
| ETV6 | HMOX1 | HMOX1 |
| IFNG | SLPI | SLPI |
| CRP | NQO1 | NQO1 |
| DNMT3A | CCL4 | CCL4 |
| CXCL8 | HMGB1 | HMGB1 |
| NBN | IL10 | IL10 |
| MIR15A | IL1RN | IL1RN |
| ABCA3 | CASP3 | CASP3 |
| GATA2 | CCL2 | CCL2 |
| FASLG | BCL2 | BCL2 |
| SFTPA1 | SCGB1A1 | SCGB1A1 |
| MIRLET7C | MFN2 | MFN2 |
| SFTPB | BAX | BAX |
| HMBS | PTGS2 | PTGS2 |
| MAP2K1 | TLR4 | TLR4 |
| MIR34B | MFN1 | MFN1 |
| TGFB1 | C3 | C3 |
| MIRLET7D | VEGFA | VEGFA |
| KMT2A | CXCL6 | CXCL6 |
| MIR17 | THBD | THBD |
| RTEL1 | XDH | XDH |
| IDH1 | CASP8 | CASP8 |
| IL1B | CAV1 | CAV1 |
| MET | FADD | FADD |
| MIR34A | TIMP2 | TIMP2 |
| MIR155 | P2RY12 | P2RY12 |
| MYC | AKAP12 | AKAP12 |
| CFTR | NOD2 | NOD2 |
| MIR145 | IL13 | IL13 |
| ROS1 | PLA2G2A | PLA2G2A |
| MIRLET7A3 | PECAM1 | PECAM1 |
| BRCA1 | ADAR | ADAR |
| NKX2-1 | GSR | GSR |
| SFTPA2 | GOT1 | GOT1 |
| MIR221 | GJA1 | GJA1 |
| MMP9 | FLT1 | FLT1 |
| NUP214 | FGA | FGA |
| VEGFA | FCGR2B | FCGR2B |
| MUC5B | FCGR2A | FCGR2A |
| SERPINA1 | FCGR1A | FCGR1A |
| MIR125A | F3 | F3 |
| MPO | F2 | F2 |
| NSD1 | ALB | ALB |
| ERCC6 | EPHX2 | EPHX2 |
| IRF1 | AGER | AGER |
| HMOX1 | CYP1A1 | CYP1A1 |
| RB1 | ADRA2A | ADRA2A |
| MIR93 | IFNG | IFNG |
| ELANE | IL2 | IL2 |
| MIR486-1 | PAWR | PAWR |
| ALB | PAK1 | PAK1 |
| CASP3 | OPA1 | OPA1 |
| MIR29A | NOS2 | NOS2 |
| BAX | MIR330 | MIR330 |
| IDH2 | MMP9 | MMP9 |
| RARA | MMP7 | MMP7 |
| CTNNB1 | MMP2 | MMP2 |
| ABL1 | MIR96 | MIR96 |
| PRKN | KDR | KDR |
| TLR4 | ITGB2 | ITGB2 |
| ACE | AQP5 | AQP5 |
| RTEL1-TNFRSF6B | IL18 | IL18 |
| MIR223 | IL6 | IL6 |
| CCL2 | IL4 | IL4 |
| MIR499A | PROCR | PROCR |
| CSF3 | MST1R | MST1R |
| PAX5 |  | EGFR |
| BCL2 |  | TP53 |
| CBFB |  | KRAS |
| TERC |  | TERT |
| F2 |  | FLT3 |
| MIR34C |  | PTEN |
| HRAS |  | MIR126 |
| MIR222 |  | CEBPA |
| ICAM1 |  | KIT |
| STAT5B |  | AKT1 |
| MIRLET7B |  | BRAF |
| CYP2A6 |  | STAT3 |
| MIR210 |  | PIK3CA |
| IL4 |  | SFTPC |
| MLLT10 |  | NPM1 |
| MIR200C |  | RUNX1 |
| MAPK1 |  | CDKN2A |
| MIR200B |  | ERBB2 |
| PTPN11 |  | MIR21 |
| BCOR |  | NRAS |
| HLA-DRB1 |  | ALK |
| MIR130A |  | JAK2 |
| NOS2 |  | RANBP2 |
| CCND1 |  | ETV6 |
| THBD |  | CRP |
| ABCB1 |  | DNMT3A |
| PML |  | CXCL8 |
| GATA1 |  | NBN |
| MIR18A |  | MIR15A |
| MIR128-2 |  | ABCA3 |
| MALAT1 |  | GATA2 |
| LCN2 |  | SFTPA1 |
| NOTCH1 |  | MIRLET7C |
| BRCA2 |  | SFTPB |
| CSF2 |  | HMBS |
| SETBP1 |  | MAP2K1 |
| MIR30D |  | MIR34B |
| SFTPD |  | MIRLET7D |
| MIR205 |  | KMT2A |
| MIR19A |  | MIR17 |
| MIR141 |  | RTEL1 |
| ATM |  | IDH1 |
| CREBBP |  | MET |
| MIR204 |  | MIR34A |
| CTLA4 |  | MIR155 |
| GSTM1 |  | MYC |
| COPA |  | CFTR |
| TET2 |  | MIR145 |
| LOC110806263 |  | ROS1 |
| MIRLET7E |  | MIRLET7A3 |
| SETD2 |  | BRCA1 |
| TLR3 |  | NKX2-1 |
| NFE2L2 |  | SFTPA2 |
| IL13 |  | MIR221 |
| MIR20A |  | NUP214 |
| MIR183 |  | MUC5B |
| SERPINE1 |  | SERPINA1 |
| MIR185 |  | MIR125A |
| IL17A |  | MPO |
| MIR200A |  | NSD1 |
| IL2 |  | ERCC6 |
| TP73 |  | IRF1 |
| HLA-B |  | RB1 |
| ZBTB16 |  | MIR93 |
| FGFR1 |  | MIR486-1 |
| IKZF1 |  | MIR29A |
| HGF |  | IDH2 |
| PTGS2 |  | RARA |
| MDM2 |  | CTNNB1 |
| CD4 |  | ABL1 |
| DDX41 |  | PRKN |
| PICALM |  | ACE |
| WT1 |  | RTEL1-TNFRSF6B |
| MIR107 |  | MIR223 |
| HFE |  | MIR499A |
| U2AF1 |  | CSF3 |
| FAS |  | PAX5 |
| MXRA5 |  | CBFB |
| CYP1A1 |  | TERC |
| MAP3K8 |  | MIR34C |
| MIR29C |  | HRAS |
| IL18 |  | MIR222 |
| MIR137 |  | ICAM1 |
| BCR |  | STAT5B |
| CHEK2 |  | MIRLET7B |
| MIR152 |  | CYP2A6 |
| LPP |  | MIR210 |
| MTHFR |  | MLLT10 |
| ITGA3 |  | MIR200C |
| MIR133B |  | MAPK1 |
| DICER1 |  | MIR200B |
| MIR98 |  | PTPN11 |
| HIF1A |  | BCOR |
| MARS1 |  | HLA-DRB1 |
| STAT1 |  | MIR130A |
| CDKN1A |  | CCND1 |
| DNAJC21 |  | ABCB1 |
| TSC2 |  | PML |
| MIR429 |  | GATA1 |
| MIR197 |  | MIR18A |
| IL1RN |  | MIR128-2 |
| SH3GL1 |  | MALAT1 |
| MIR203A |  | LCN2 |
| PARN |  | NOTCH1 |
| VWF |  | BRCA2 |
| ELN |  | CSF2 |
| CCR6 |  | SETBP1 |
| ENO2 |  | MIR30D |
| CD274 |  | MIR205 |
| CCL11 |  | MIR19A |
| JUN |  | MIR141 |
| DSP |  | ATM |
| CAV1 |  | CREBBP |
| LRRC56 |  | MIR204 |
| ACE2 |  | CTLA4 |
| STAR |  | GSTM1 |
| MIF |  | COPA |
| GNB1 |  | TET2 |
| MEFV |  | LOC110806263 |
| CDH1 |  | MIRLET7E |
| FCGR2A |  | SETD2 |
| PDGFRB |  | TLR3 |
| MIRLET7G |  | MIR20A |
| GSTP1 |  | MIR183 |
| MIR16-1 |  | MIR185 |
| TRAF3 |  | MIR200A |
| APOE |  | TP73 |
| STK11 |  | HLA-B |
| FAM13A |  | ZBTB16 |
| CDK4 |  | FGFR1 |
| IRF3 |  | IKZF1 |
| EDN1 |  | HGF |
| NTRK1 |  | MDM2 |
| GPT |  | CD4 |
| CXCR4 |  | DDX41 |
| SOD1 |  | PICALM |
| CDKN1B |  | WT1 |
| CFH |  | MIR107 |
| IL1A |  | HFE |
| HP |  | U2AF1 |
| SPP1 |  | MXRA5 |
| MYB |  | MAP3K8 |
| LPIN1 |  | MIR29C |
| CPT2 |  | MIR137 |
| PLG |  | BCR |
| INPP5E |  | CHEK2 |
| HMGB1 |  | MIR152 |
| MYH11 |  | LPP |
| TLR2 |  | MTHFR |
| EGF |  | ITGA3 |
| CDKN2B |  | MIR133B |
| RARB |  | DICER1 |
| MTOR |  | MIR98 |
| SERPINA3 |  | HIF1A |
| BCL2L1 |  | MARS1 |
| ABCC1 |  | STAT1 |
| SMAD4 |  | CDKN1A |
| MMP2 |  | DNAJC21 |
| ALAD |  | TSC2 |
| CXCL10 |  | MIR429 |
| F3 |  | MIR197 |
| CCL5 |  | SH3GL1 |
| KDR |  | MIR203A |
| NFKB1 |  | PARN |
| IL2RA |  | VWF |
| ITGAM |  | ELN |
| MIR146A |  | CCR6 |
| HLA-A |  | ENO2 |
| EP300 |  | CD274 |
| MAPK8 |  | CCL11 |
| FGF2 |  | JUN |
| NOS3 |  | DSP |
| FGF10 |  | LRRC56 |
| SCGB1A1 |  | ACE2 |
| F5 |  | STAR |
| B2M |  | MIF |
| VHL |  | GNB1 |
| PARP1 |  | MEFV |
| CYCS |  | CDH1 |
| MIR320A |  | PDGFRB |
| CD36 |  | MIRLET7G |
| NPPB |  | GSTP1 |
| DPP9 |  | MIR16-1 |
| FLT1 |  | TRAF3 |
| PRTN3 |  | APOE |
| NLRP3 |  | STK11 |
| FIP1L1 |  | FAM13A |
| STN1 |  | CDK4 |
| MUC1 |  | IRF3 |
| SELP |  | NTRK1 |
| ALOX5 |  | GPT |
| CD8A |  | CXCR4 |
| ERBB3 |  | SOD1 |
| ASXL1 |  | CDKN1B |
| NQO1 |  | CFH |
| PMS2 |  | IL1A |
| HBB |  | HP |
| PPARG |  | SPP1 |
| CCL3 |  | MYB |
| FGFR3 |  | LPIN1 |
| MPL |  | CPT2 |
| INS |  | PLG |
| RUNX1T1 |  | INPP5E |
| MIR143 |  | MYH11 |
| ATP11A |  | TLR2 |
| TIMP1 |  | EGF |
| PRKAR1A |  | CDKN2B |
| SAA1 |  | RARB |
| FLCN |  | MTOR |
| MAPK3 |  | SERPINA3 |
| CASP9 |  | BCL2L1 |
| SERPINC1 |  | ABCC1 |
| TSC1 |  | SMAD4 |
| MVP |  | ALAD |
| CYP2C19 |  | CXCL10 |
| TBK1 |  | CCL5 |
| CD46 |  | NFKB1 |
| ADRB2 |  | IL2RA |
| FN1 |  | ITGAM |
| FBN1 |  | MIR146A |
| ERCC2 |  | HLA-A |
| XRCC1 |  | EP300 |
| NUMA1 |  | MAPK8 |
| TNFRSF1A |  | FGF2 |
| SRC |  | NOS3 |
| FBXW7 |  | FGF10 |
| IL5 |  | F5 |
| XIAP |  | B2M |
| FOXP3 |  | VHL |
| FGFR2 |  | PARP1 |
| EPO |  | CYCS |
| CXCL12 |  | MIR320A |
| CHAT |  | CD36 |
| IRF2BP2 |  | NPPB |
| BCL10 |  | DPP9 |
| C3 |  | PRTN3 |
| TGFBR2 |  | NLRP3 |
| CD34 |  | FIP1L1 |
| RET |  | STN1 |
| FARSB |  | MUC1 |
| NOD2 |  | SELP |
| IL3 |  | ALOX5 |
| PDCD1 |  | CD8A |
| MB |  | ERBB3 |
| KNG1 |  | ASXL1 |
| BDNF |  | PMS2 |
| ERCC6L2 |  | HBB |
| JAK3 |  | CCL3 |
| NF1 |  | FGFR3 |
| TRMU |  | MPL |
| PIK3CG |  | INS |
| G6PD |  | RUNX1T1 |
| TTR |  | MIR143 |
| MME |  | ATP11A |
| CP |  | TIMP1 |
| MIR130B |  | PRKAR1A |
| SEPTIN9 |  | SAA1 |
| MCL1 |  | FLCN |
| IGF1 |  | MAPK3 |
| HLA-DQB1 |  | CASP9 |
| NCAM1 |  | SERPINC1 |
| RYR1 |  | TSC1 |
| TGM6 |  | MVP |
| TYMS |  | CYP2C19 |
| CD44 |  | TBK1 |
| ADAMTS13 |  | CD46 |
| MIR27A |  | ADRB2 |
| FGF7 |  | FN1 |
| REN |  | FBN1 |
| RAF1 |  | ERCC2 |
| SOD2 |  | XRCC1 |
| PDGFRA |  | NUMA1 |
| APC |  | TNFRSF1A |
| MBP |  | SRC |
| MBL2 |  | FBXW7 |
| CCN2 |  | IL5 |
| PTPRC |  | XIAP |
| ANXA5 |  | FOXP3 |
| ENG |  | FGFR2 |
| CEACAM6 |  | EPO |
| ABCG2 |  | CXCL12 |
| PMP22 |  | CHAT |
| BIRC5 |  | IRF2BP2 |
| FHIT |  | BCL10 |
| IL1R1 |  | TGFBR2 |
| MAPK14 |  | CD34 |
| MEG3 |  | RET |
| CALCA |  | FARSB |
| LEP |  | IL3 |
| SYK |  | PDCD1 |
| TBL1XR1 |  | MB |
| SMPD1 |  | KNG1 |
| IGF1R |  | BDNF |
| IL7 |  | ERCC6L2 |
| CDK2 |  | JAK3 |
| FOS |  | NF1 |
| TICAM1 |  | TRMU |
| EZH2 |  | PIK3CG |
| ESR1 |  | G6PD |
| TOP1 |  | TTR |
| MIR142 |  | MME |
| THPO |  | CP |
| CCR5 |  | MIR130B |
| CD79A |  | SEPTIN9 |
| AGER |  | MCL1 |
| RHOA |  | IGF1 |
| NUP98 |  | HLA-DQB1 |
| CST3 |  | NCAM1 |
| TNFSF10 |  | RYR1 |
| CREB1 |  | TGM6 |
| SMARCA4 |  | TYMS |
| H2AC18 |  | CD44 |
| KITLG |  | ADAMTS13 |
| CHUK |  | MIR27A |
| CYP3A4 |  | FGF7 |
| IL6ST |  | REN |
| PLAT |  | RAF1 |
| DNMT1 |  | SOD2 |
| SHH |  | PDGFRA |
| IFNA1 |  | APC |
| HAVCR1 |  | MBP |
| MIR144 |  | MBL2 |
| BCL6 |  | CCN2 |
| CAT |  | PTPRC |
| MIR150 |  | ANXA5 |
| SMAD3 |  | ENG |
| CTSB |  | CEACAM6 |
| NGF |  | ABCG2 |
| MYCN |  | PMP22 |
| GBA1 |  | BIRC5 |
| NPPA |  | FHIT |
| SLC2A1 |  | IL1R1 |
| STIL |  | MAPK14 |
| ALDH2 |  | MEG3 |
| ANGPT2 |  | CALCA |
| RELA |  | LEP |
| CYP2E1 |  | SYK |
| ADA |  | TBL1XR1 |
| VCAM1 |  | SMPD1 |
| AGTR1 |  | IGF1R |
| HSP90AA1 |  | IL7 |
| EDNRA |  | CDK2 |
| GZMB |  | FOS |
| ITGB2 |  | TICAM1 |
| PIK3C2A |  | EZH2 |
| SLC9A3 |  | ESR1 |
| MRTFA |  | TOP1 |
| HPRT1 |  | MIR142 |
| MECOM |  | THPO |
| HADHA |  | CCR5 |
| MMP1 |  | CD79A |
| RINT1 |  | RHOA |
| SELE |  | NUP98 |
| CALR |  | CST3 |
| TNFRSF10B |  | TNFSF10 |
| SOX9 |  | CREB1 |
| CXCR3 |  | SMARCA4 |
| MIR140 |  | H2AC18 |
| GAPDH |  | KITLG |
| MIR199A1 |  | CHUK |
| MLH1 |  | CYP3A4 |
| FGA |  | IL6ST |
| BMPR2 |  | PLAT |
| SIRT1 |  | DNMT1 |
| STAT5A |  | SHH |
| MSH2 |  | IFNA1 |
| CD40LG |  | HAVCR1 |
| ERCC1 |  | MIR144 |
| FOXO3 |  | BCL6 |
| JAK1 |  | MIR150 |
| SF3B1 |  | SMAD3 |
| LBP |  | CTSB |
| MIR30A |  | NGF |
| CDK1 |  | MYCN |
| CD40 |  | GBA1 |
| ICOSLG |  | NPPA |
| CEBPB |  | SLC2A1 |
| MUC5AC |  | STIL |
| CSF1 |  | ALDH2 |
| ANGPT1 |  | ANGPT2 |
| TLR5 |  | RELA |
| NOTCH3 |  | CYP2E1 |
| NABP1 |  | ADA |
| ITGB1 |  | VCAM1 |
| SLC22A12 |  | AGTR1 |
| GFAP |  | HSP90AA1 |
|  |  | EDNRA |
|  |  | GZMB |
|  |  | PIK3C2A |
|  |  | SLC9A3 |
|  |  | MRTFA |
|  |  | HPRT1 |
|  |  | MECOM |
|  |  | HADHA |
|  |  | MMP1 |
|  |  | RINT1 |
|  |  | SELE |
|  |  | CALR |
|  |  | TNFRSF10B |
|  |  | SOX9 |
|  |  | CXCR3 |
|  |  | MIR140 |
|  |  | GAPDH |
|  |  | MIR199A1 |
|  |  | MLH1 |
|  |  | BMPR2 |
|  |  | SIRT1 |
|  |  | STAT5A |
|  |  | MSH2 |
|  |  | CD40LG |
|  |  | ERCC1 |
|  |  | FOXO3 |
|  |  | JAK1 |
|  |  | SF3B1 |
|  |  | LBP |
|  |  | MIR30A |
|  |  | CDK1 |
|  |  | CD40 |
|  |  | ICOSLG |
|  |  | CEBPB |
|  |  | MUC5AC |
|  |  | CSF1 |
|  |  | ANGPT1 |
|  |  | TLR5 |
|  |  | NOTCH3 |
|  |  | NABP1 |
|  |  | ITGB1 |
|  |  | SLC22A12 |
|  |  | GFAP |
