# Supplementary material for: Uncovering the active constituents and mechanisms of Rujin Jiedu powder for ameliorating LPS-induced acute lung injury using network pharmacology and experimental investigations
Source: Front Pharmacol. 2023 May 11;14:1186699. doi: 10.3389/fphar.2023.1186699 (PMC10210165; doi:10.3389/fphar.2023.1186699)
Supplement: Supplementary file 2 [file DataSheet3.docx]

Table S3 | Active compounds and targets information of 6 herbs in RJJD

| **Herb Attribution** | **Number** | **Compound** | **Target** |
| --- | --- | --- | --- |
| Gancao | GC1 | Glabrone | NOS2, PTGS1, F2, ESR1, AR, SCN5A, PPARG, F10, PTGS2, RXRA, ACHE, ESR2, DPP4, MAPK14, GSK3B, CDK2, CHEK1, PRSS1, PIM1, CCNA2, CAM |
| Gancao | GC10 | Formononetin | NOS2, PTGS1, CHRM1, ESR1, AR, PPARG, PTGS2, RXRA, PDE3A, ADRA1A, SLC6A3, ADRB2, SLC6A4, ESR2, DPP4, MAPK14, GSK3B, HSP90, CDK2, MAOB, CHEK1, PRKACA, PRSS1, PIM1, CCNA2, CAM, PKIA, F2, NOS3, ACHE, JUN, PPARG, IL4, SIRT1, ATP5F1B, MT-ND6, HSD3B2, HSD3B1 |
| Gancao | GC11 | Echinatin | NOS2, PTGS1, ESR1, PPARG, PTGS2, RXRA, PDE3A, MAPK14, GSK3B, HSP90, CDK2, LACTB, MAOB, CHEK1, PRKACA, PIM1, CCNA2, NCOA2, PKIA |
| Gancao | GC12 | Isoliquiritigenin | PTGS1, ESR1, PPARG, PTGS2, CA2, ADRB2, ESR2, MAPK14, GSK3B, HSP90, CDK2, MAOB, PRKACA, PKIA, NOS2, AR, PIK3CG, LACTB, PIM1, CCNA2, NCOA2, FOS, BAX, SELE, VCAM1, GABBR1, TYR, F11R, JAK2, MT2A, SLC2A1 |
| Gancao | GC13 | Glycycoumarin | ACHE, PTPN1, PDE4D, CCR4 |
| Gancao | GC14 | Licoisoflavone B | NOS2, F2, ESR1, AR, PPARG, F10, PTGS2, ACHE, TOP2, ESR2, GSK3B, CDK2, CHEK1, PRSS1, PIM1, CCNA2, CAM |
| Gancao | GC15 | Gancaonin I | PPARG, ESR1, MTOR, PIK3CA |
| Gancao | GC16 | Licoisoflavone A or Licoflavonol | PTPN1, CYP19A1, ACHE, ESR1 |
| Gancao | GC17 | Licochalcone A | NOS2, PTGS1, CHRM1, ESR1, AR, SCN5A, PPARG, F10, PTGS2, CA2, ADRA1B, SLC6A3, ESR2, MAPK14, GSK3B, HSP90, CDK2, CHEK1, PIM1, CCNA2, CAM, ADRB2, NCOA2, RELA, STAT3, CCND1, BCL2, EIF6, MAPK1, RB1, CDK4, FOSL2 |
| Gancao | GC18 | glycyrrhizic acid | HSD11B2, HSD11B1 |
| Gancao | GC2 | Phloretic acid | ESR2, FYN, LCK, EGFR, ESR1, KDM4E |
| Gancao | GC3 | Neoliquiritin | F10, F7, CAM |
| Gancao | GC4 | Liquiritigenin | PTGS1, ESR1, PTGS2, RXRA, ADRB2, HSP90, PIK3CG, PRKACA, LACTB, MAOB, SLC6A4, PKIA |
| Gancao | GC5 | Ononin | NOS2, F2, ESR1, AR, PTGS2, F7, KDR, DPP4, CAM, PPARG |
| Gancao | GC6 | licochalcone B | NOS2, PTGS1, ESR1, AR, PPARG, PTGS2, CA2, PDE3A, ADRB2, ESR2, MAPK14, GSK3B, HSP90, CDK2, CHEK1, PRKACA, PIM1, CCNA2, CAM |
| Gancao | GC7 | Homobutein | APP, AKR1B1, ABCG2, EGFR, TERT, NOS2, BACE1, TUBB1, PTGS2 |
| Gancao | GC8 | Daidzein | PTGS1, ESR1, PPARG, PTGS2, RXRA, ADRB2, MAPK14, HSP90, CDK2, CHEK1, PRKACA, PRSS1, PDE3A, RELA, VEGFA, FOS, CDKN1A, EIF6, BAX, TNF, JUN, IL6, NOS2, CASP3, TP53, LDLR, CAT, IGF1R, STAT1, PPARG, CYP3A4, CAV1, ICAM1, APOB, MTTP, VCAM1, NOS3, ECE1, GABBR1, IL4, CPT1A, CAM, AHR, RHOA, MT2A, FCER2, ATP5F1B, MT-ND6, HSD3B2, HSD3B1, TFF1, TRPM2, GH1, IGF1, GHR, TGFB1I1, CD5L, CYP21, GADD45A, BARD1, RBM45, BAP1, RAD51 |
| Gancao | GC9 | Glycyroside | IL2, TNF, ALDH2 |
| Huangbai | HB1 | 4-Methoxy-N-methyl-2-quinolone | CCNE2, CDK2, CCNE1, KCNA3, KDR, CYP19A1 |
| Huangbai | HB2 | Kumatakenin | NOS2, PTGS1, AR, SCN5A, PTGS2, ESR2, DPP4, HSP90, CDK2, CHEK1, PRSS1, NCOA2, CAM |
| Huanglian | HL1 | Boldine | PTGS1, Chrm3, CHRM1, AR, SCN5A, CHRM5, PTGS2, CHRM4, RXRA, OPRD1, ACHE, ADRA1B, SLC6A3, ADRB2, ADRA1D, TOP2, SLC6A4, OPRM1, HSP90 |
| Huanglian | HL2 | Berberrubine | NOS2, PTGS1, KCNH2, ESR1, AR, SCN5A, PTGS2, NOS3, RXRA, PRKACA, PRSS1, NCOA2, CAM |
| Huanglian | HL3 | Coptisine | NOS2, PTGS1, KCNH2, ESR1, AR, SCN5A, PTGS2, NOS3, PRSS1 |
| Huanglian | HL4 | Worenine | NOS2, PTGS1, ESR1, AR, PTGS2, CHEK1, PIM1 |
| Huanglian | HL5 | Acaciin | TNF, AKR1B1, IL2, ADORA1, XDH, NMUR2 |
| Huanglian | HL6 | Alpinetin | ABCG2, CYP19A1, CYP1B1, SHBG, MAOB, HSD17B1 |
| Huangqin | HQ1 | Epicatechin | PTGS1, ESR1, PTGS2, HSP90, LACTB, PRKACA |
| Huangqin | HQ2 | Viscidulin III | NOS2, F2, AR, SCN5A, F10, PTGS2, F7, TOP2, DPP4, HSP90, PRSS1, NCOA2, CAM |
| Huangqin | HQ3 | Norwogonin | NOS2, PTGS1, AR, PPARG, PTGS2, PDE3A, DPP4, HSP90, CDK2, PIK3CG, CHEK1, PRKACA |
| Huangqin | HQ4 | Baicalin | F10, PTPN1 |
| Huangqin | HQ5 | Chrysin-7-O-β-D-glucuronide | F2, F10, PTGS2, F7, PTPN1 |
| Huangqin | HQ6 | Baicalein | PTGS1, AR, PTGS2, HSP90, PRKACA, DPP4, PIK3CG, PDE3A, PRSS1, NCOA2, NCOA1, CAM, RELA, AKT1, VEGFA, BCL2, FOS, BAX, MMP9, CASP3, TP53, HIF1A, FOSL1, FOSL2, CDC2, CCNB1, MPO, AHR, IGF2, CYCS, ALOX12, NFATC1, TDRD7, EGLN1, NOX5, FABP5, APOD |
| Jiegeng | JG1 | Luteolin | PTGS1, AR, PTGS2, HSP90, PRSS1, NCOA2, PRKACA, DPP4, PIK3CG, RELA, EGFR, AKT1, VEGFA, CCND1, BCL2L1, CDKN1A, CASP9, MMP2, MMP9, MAPK1, IL10, RB1, CDK4, TNF, JUN, IL6, CASP3, TP53, NFKBIA, XDH, TOP1, MDM2, APP, MMP1, PCNA, ERBB2, PPARG, HMOX1, CASP7, ICAM1, MCL1, BIRC5, IL2, CCNB1, TYR, IFNG, IL4, TOP2A, GSTP1, BIRC4, SLC2A4, INSR, CD40LG, PTGES, NUF2, ADCY2, MET |
| Jiegeng | JG2 | Oroxylin A | NOS2, PTGS1, AR, SCN5A, PTGS2, RXRA, PDE3A, ADRA1B, ADRB2, DPP4, HSP90, PIK3CG, PRKACA, PRSS1, NCOA1, CAM, NCOA2, PKIA, BCL2, IL6, CASP3, CDC2, CYP1A2, CCNB1, CYP2C9 |
| Jiegeng | JG3 | platycogenic acid A | AKR1B10, CD81, PTPN1, POLB |
| Zhizi | ZZ1 | Geniposidic acid | CA2, F2 |
| Zhizi | ZZ2 | Geniposide | CA2, BCL2, HMOX1, GAP43, PLB1, GCG, GSTM1, GSTM2 |
| Zhizi | ZZ3 | Genipin | PTGS1, PTGS2, CA2, GABRA2, GABRA1, TK1, PRSS1, GRIA2, GABRA6 |
| Zhizi | ZZ4 | Jasminoside A | IL2, HSD11B2, SLC5A2, HSD11B1 |
| Zhizi | ZZ5 | Jasminoside E | PTPN1, SLC5A2, HSD11B2, PPP1CC |
| Zhizi | ZZ6 | Jasminodiol | TNF, NOS2, NPC1L1, RORA, SHBG, CYP17A1 |
| Zhizi | ZZ7 | Crocetin | CHRM3, CHRM1, GABRA2, GABRA5, ADRA1A, GABRA3, CHRM2, ADRA1B, GABRA1, IGHG1, PTGS2, NCOA2, VCAM1 |
| Zhizi/Huangqin/Huanglian/Huangbai | GY1 | Quinic acid | PTGS2, IGHG1 |
| Huanglian/Huangbai | GY2 | Higenamine | DRD2, ADRB2, ADRB1, DRD3, DRD4 |
| Gancao/Huangqin/Huanglian/Huangbai/Zhizi | GY3 | Quercetin | PTGS1, AR, PPARG, PTGS2, HSP90, PIK3CG, NCOA2, DPP4, AKR1B10, PRSS1, TOP2, F2, KCNH2, SCN5A, F10, ADRB2, MMP3, PRKACA, F7, NOS3, RXRA, ACHE, GABRA1, MAOB, RELA, EGFR, AKT1, VEGFA, CCND1, BCL2, BCL2L1, FOS, CDKN1A, EIF6, BAX, CASP9, PLAU, MMP2, MMP9, MAPK1, IL10, EGF, RB1, TNF, JUN, IL6, CDKN2A, AHSA1, CASP3, TP53, ELK1, NFKBIA, POR, ODC1, XDH, CASP8, TOP1, RAF1, SOD1, PRKCA, MMP1, HIF1A, STAT1, RUNX1T1, ALOX5, HSPA5, ERBB2, PPARG, ACACA, HMOX1, CYP3A4, CYP1A2, CAV1, MYC, F3, GJA1, CYP1A1, ICAM1, IL1B, CCL2, SELE, VCAM1, PTGER3, CXCL8, PRKCB, BIRC5, DUOX2, NOS3, HSPB1, TGFB1, MGAM, IL2, NR1I2, CYP1B1, CCNB1, PLAT, THBD, SERPINE1, COL1A1, IFNG, ALOX5, PTEN, IL1A, MPO, TOP2A, NCF1, ABCA2, HAS2, GSTP1, NFE2L2, NQO1, PARP1, AHR, PSMD3, SLC2A4, COL8A1, GYRB, CXCL11, CXCL2, DCAF5, NR1I3, CHEK2, INSR, CLDN4, PPARA, PPARD, HSF1, CRP, CXCL10, CHUK, SPP1, RUNX2, RASSF1, E2F1, E2F2, ACP3, CTSD, IGFBP3, IGF2, CD40LG, IRF1, ERBB3, PON1, DIO1, PCOLCE, NPEPPS, HK2, NKX3-1, RASA1, PRXC1A, GSTM1, GSTM2 |
| Gancao/Huangqin/Zhizi | GY4 | kaempferol | NOS2, PTGS1, AR, PPARG, PTGS2, HSP90, PIK3CG, PRKACA, NCOA2, DPP4, PRSS1, PGR, F2, CHRM1, NOS3, GABRA2, ACHE, SLC6A2, CHRM2, ADRA1B, GABRA1, TOP2, F7, CAM, RELA, IKBKB, AKT1, BCL2, BAX, TNF, JUN, AHSA1, CASP3, MAPK8, XDH, MMP1, STAT1, ALOX5, PPARG, HMOX1, CYP3A4, CYP1A2, CYP1A1, ICAM1, SELE, VCAM1, NR1I2, CYP1B1, ALOX5, HAS2, GSTP1, AHR, PSMD3, SLC2A4, NR1I3, INSR, DIO1, PPP3CA, PRXC1A, GSTM1, GSTM2, AKR1C3, SLPI |
| Huanglian/Huangbai | GY5 | Demethyleneberb-erine | ACHE, SIGMAR1, CDC42, RAC1, HTR2B, ADRA2B |
| Huanglian/Huangbai | GY6 | Jatrorrhizine | NOS2, PTGS1, KCNH2, ESR1, AR, SCN5A, PTGS2, NOS3, RXRA, ESR2, HSP90, CDK2, PRSS1, PIM1, CCNA2, NCOA2, CAM, KCNMA1, TOP2, PRKACA |
| Huanglian/Huangbai | GY7 | Palmatine | NOS2, PTGS1, KCNH2, ESR1, AR, SCN5A, PTGS2, NOS3, RXRA, ADRB2, ESR2, HSP90, PRSS1, PIM1, NCOA2, CAM, PRKACA, CDK2, F7 |
| Huanglian/Huangbai | GY8 | Limonin | CYP3A4 |
| Huanglian/Huangbai | GY9 | Obacunone | OPRK1, MIF, OPRM1, OPRD1, MAPK1, CTSS |
