# Supplementary figures and images for: Uncovering the active constituents and mechanisms of Rujin Jiedu powder for ameliorating LPS-induced acute lung injury using network pharmacology and experimental investigations
Source: Front Pharmacol. 2023 May 11;14:1186699. doi: 10.3389/fphar.2023.1186699 (PMC10210165; doi:10.3389/fphar.2023.1186699)

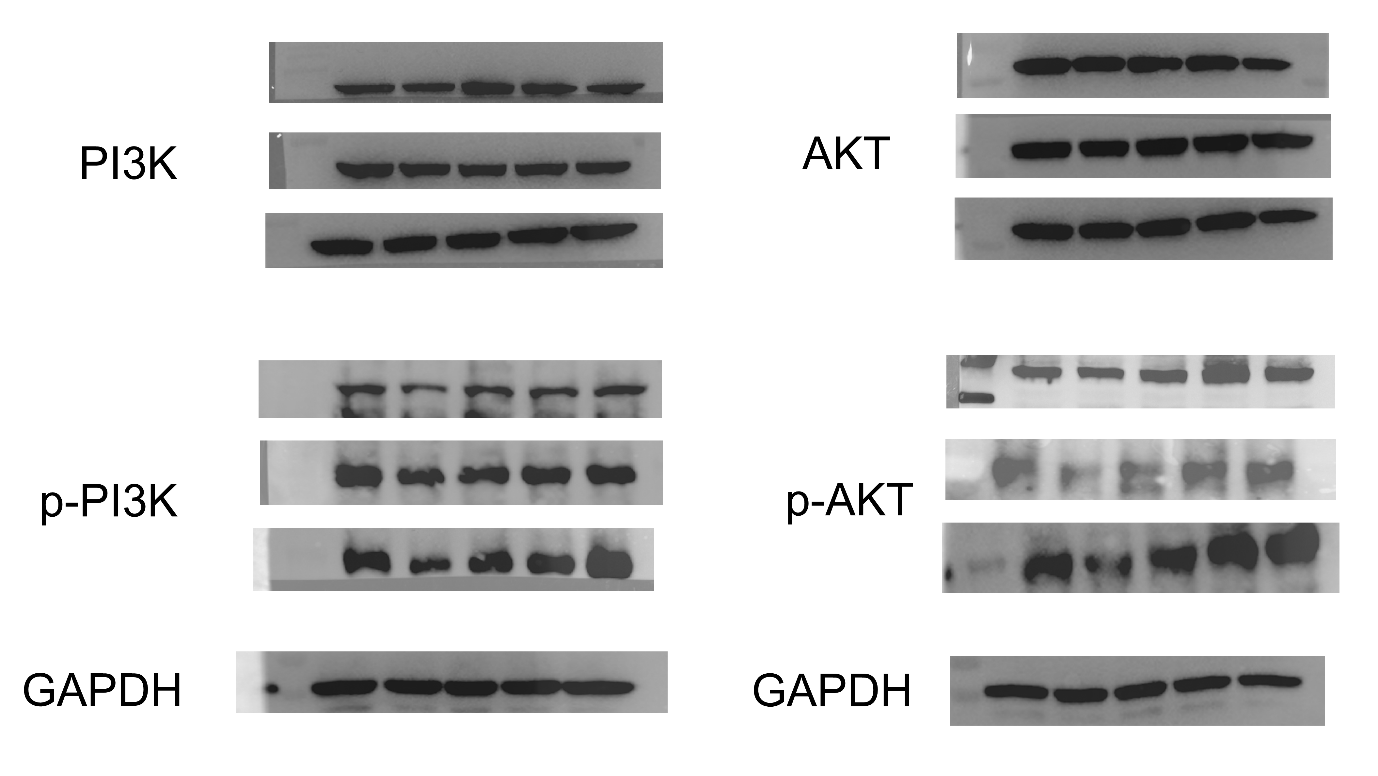


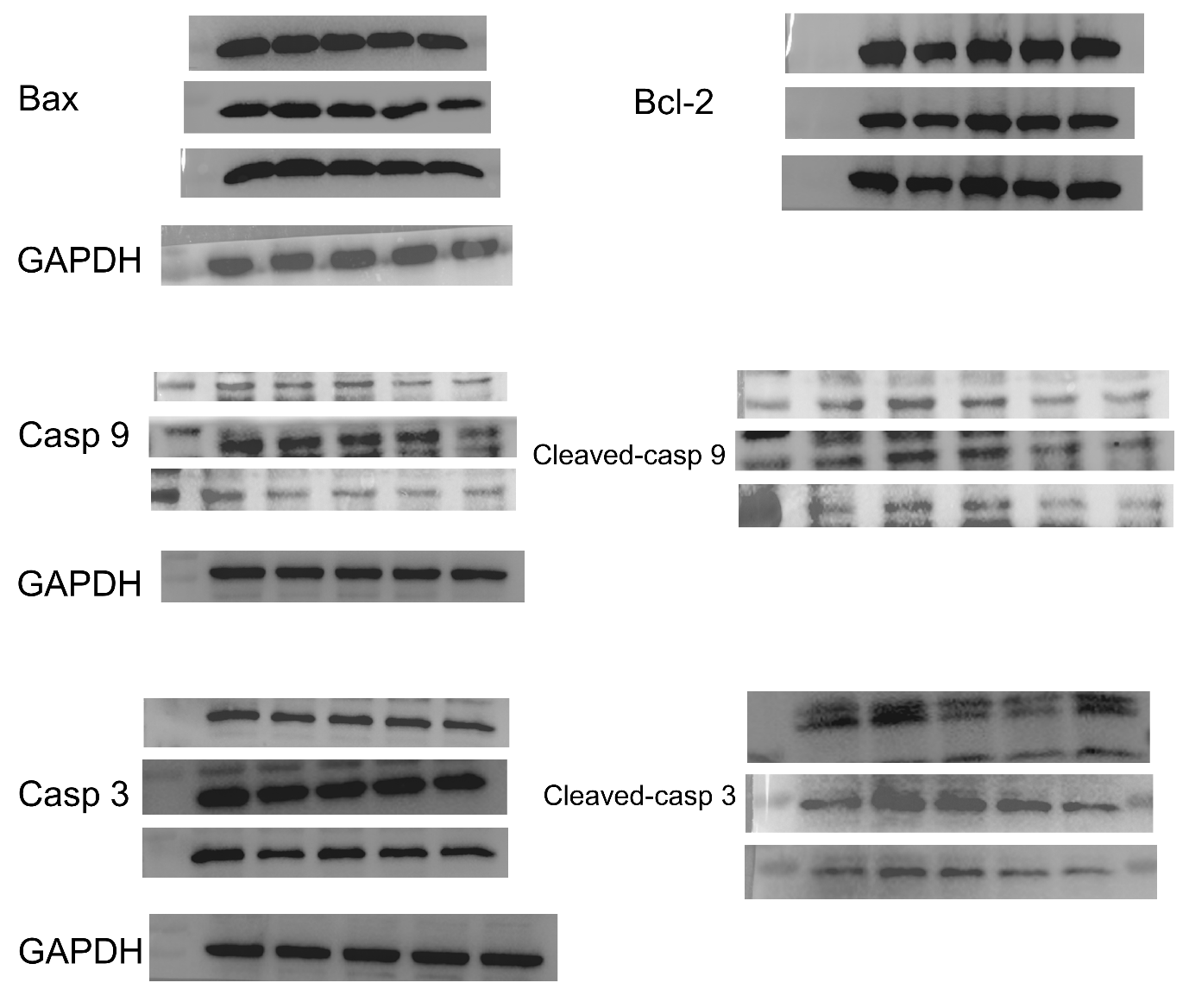


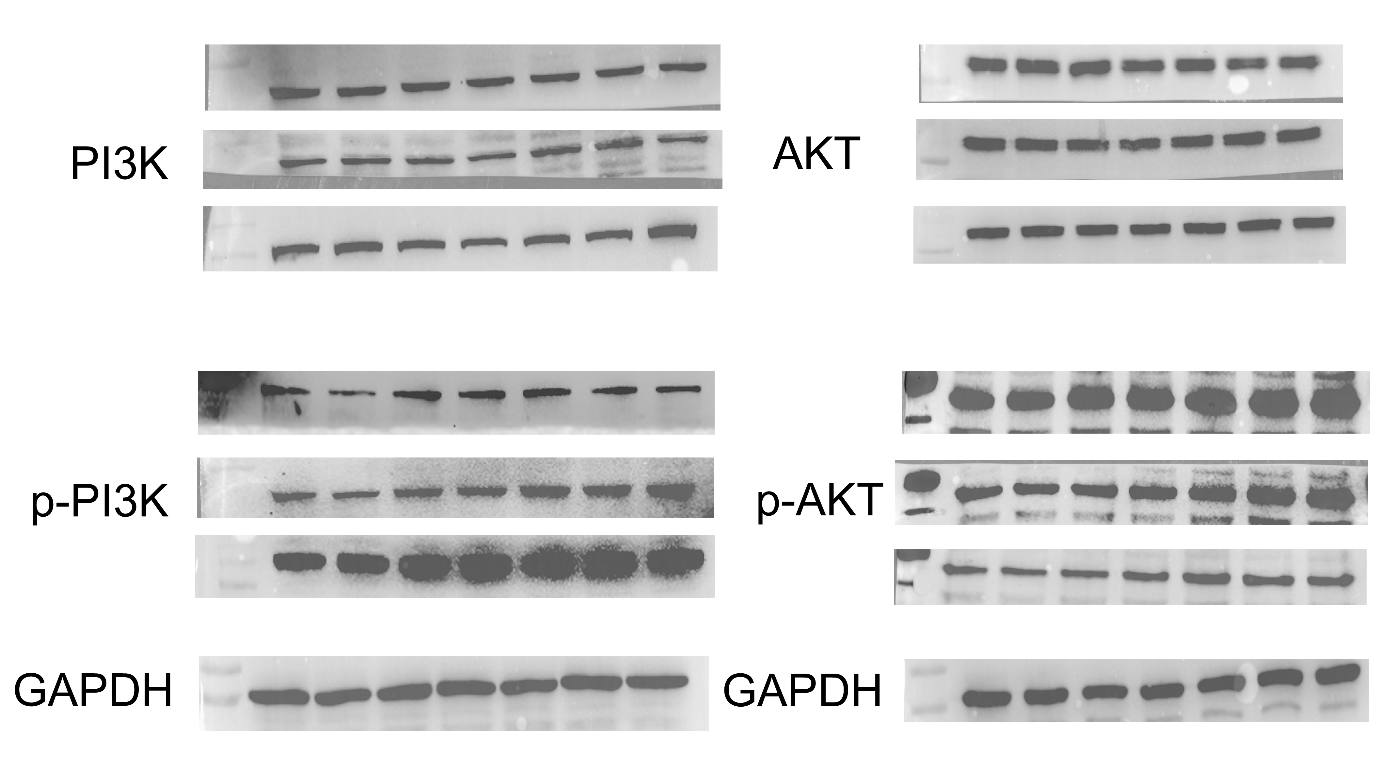


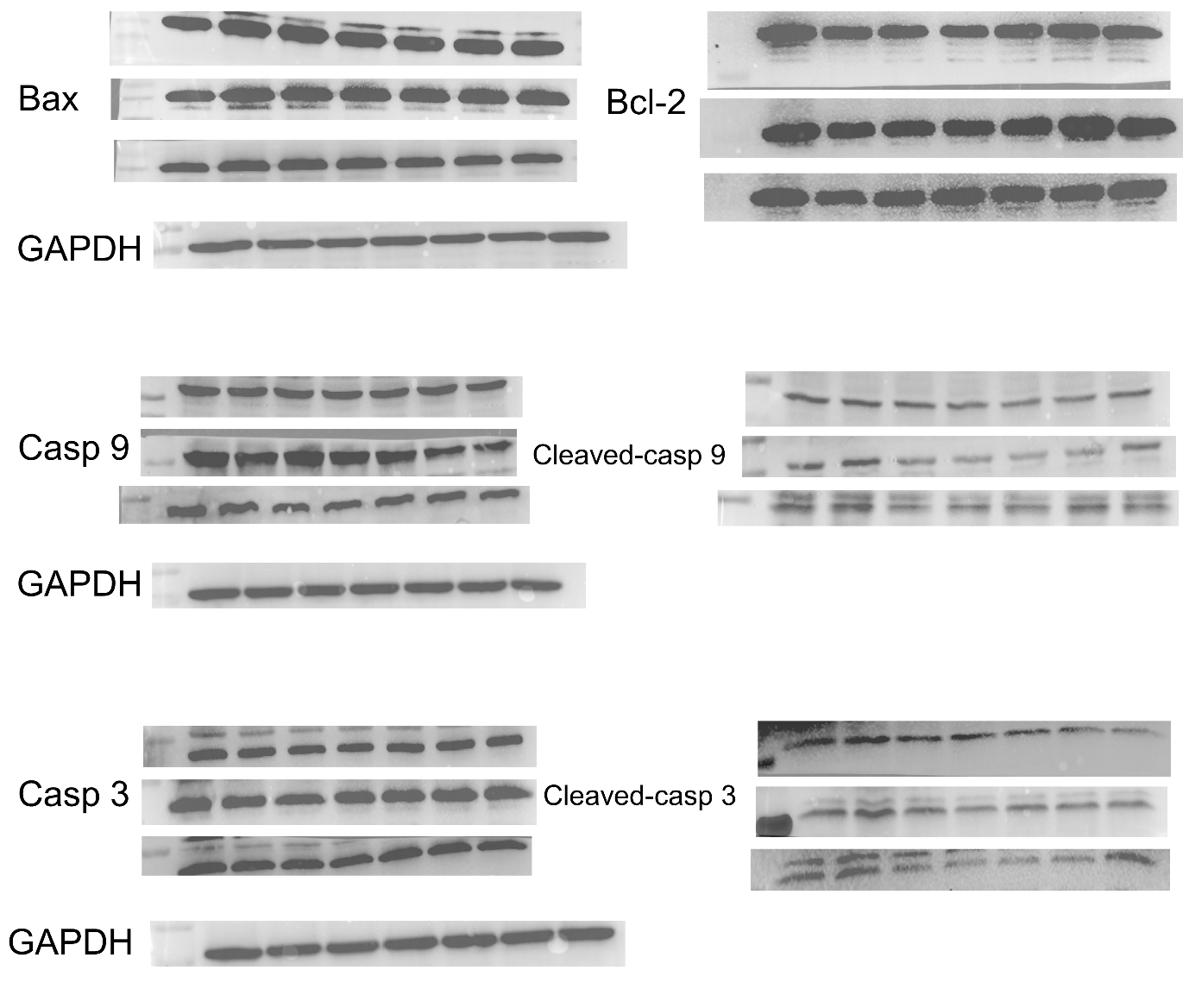

Supplement: Supplementary file 3 [file DataSheet5.docx]
