# Supplementary material for: Uncovering the active constituents and mechanisms of Rujin Jiedu powder for ameliorating LPS-induced acute lung injury using network pharmacology and experimental investigations
Source: Front Pharmacol. 2023 May 11;14:1186699. doi: 10.3389/fphar.2023.1186699 (PMC10210165; doi:10.3389/fphar.2023.1186699)
Supplement: Supplementary file 4 [file DataSheet2.docx]

Figure S2 | Total ion flow diagram of RJJD in negative ion mode and positive ion mode.

Table S2 | The chemical components of RJJD

| **NO** | **RT/min** | **Ion model** | **Measured mass /Da** | **Calculated mass /Da** | **Error /ppm** | **Molecular formula** | **Identification** | **Source** | **Peak Area** |
| --- | --- | --- | --- | --- | --- | --- | --- | --- | --- |
|  | 0.79 | [M+H]^+^ | 147.1127 | 147.1128 | -0.388 | C_6_H_14_N_2_O_2_ | L-Lysine | M.H. | 10810505 |
|  | 0.86 | [M+H]^+^ | 104.1073 | 104.107 | 3.41 | C_5_H_14_O_N_ | γ-Aminobutyric acid | M.H. | 1519896028 |
|  | 0.88 | [M+COOH]^-^ | 195.0503 | 195.0499 | 1.709 | C_5_H_10_O_5_ | Arabinose | M.H. | 1389186106 |
|  | 0.89 | [M+H]^+^ | 175.1189 | 175.119 | -0.584 | C_6_H_15_O_2_N_4_ | L-Arginine | M.H. | 11154970129 |
|  | 0.91 | [M+COOH]^-^ | 711.2211 | 711.219 | 2.962 | C_24_H_42_O_21_ | Tetrasaccharide | M.H. | 162519469 |
|  | 0.91 | [M-H]^-^ | 191.0553 | 191.055 | 1.243 | C_7_H_12_O_6_ | Quinic acid | M.H. | 3856060596 |
|  | 0.92 | [M+H]^+^ | 116.0708 | 116.0706 | 1.949 | C_5_H_9_NO_2_ | L-Proline | M.H. | 5772839849 |
|  | 0.94 | [M-H]^-^ | 341.109 | 341.1078 | 3.524 | C_12_H_22_O_11_ | Sucrose | M.H. | 2486889113 |
|  | 0.94 | [M-H]^-^ | 179.0551 | 179.055 | 0.73 | C_6_H_12_O_6_ | Fructose | HL/HB | 520319183 |
|  | 0.97 | [M+COOH]^-^ | 549.1678 | 549.1661 | 2.876 | C_18_H_32_O_16_ | Trisaccharide | M.H. | 192178799 |
|  | 0.97 | [M-H]- | 503.162 | 503.1607 | 2.641 | C_18_H_32_O_16_ | Inulin | JG | 128879077 |
|  | 1.04 | [M-H]^-^ | 133.0129 | 133.0131 | -1.696 | C_4_H_6_O_5_ | Malic acid | M.H. | 5931871190 |
|  | 1.05 | [M+H]^+^ | 136.0617 | 136.0618 | -0.381 | C_5_H_5_N_5_ | Adenine | M.H. | 2910580798 |
|  | 1.05 | [M+H]^+^ | 244.0926 | 244.0928 | -0.643 | C_9_H_13_N_3_O_5_ | Cytidine | M.H. | 233744996 |
|  | 1.12 | [M+H]^+^ | 166.0862 | 166.0863 | -0.259 | C_9_H_11_NO_2_ | D-Phenylalanine | HL | 557438940 |
|  | 1.15 | [M+H]^+^ | 118.0865 | 118.0863 | 2.026 | C_5_H_11_NO_2_ | Valine | M.H. | 600404304 |
|  | 1.25 | [M+H]^+^ | 124.0395 | 124.0393 | 1.428 | C_6_H_5_NO_2_ | Nicotinic acid | M.H. | 176084894 |
|  | 1.27 | [M-H]^-^ | 191.0189 | 191.0188 | 1.288 | C_6_H_8_O_7_ | Citric acid | M.H. | 15973243498 |
|  | 1.28 | [M+H]^+^ | 137.0458 | 137.0458 | 0.143 | C_5_H_4_N_4_O | Hypoxanthine | M.H. | 52384750 |
|  | 1.28 | [M+H]^+^ | 182.0811 | 182.0812 | -0.213 | C_9_H_11_NO_3_ | L-Tyrosine | M.H. | 206758033 |
|  | 1.55 | [M+H]^+^ | 113.0348 | 113.0349 | 2.56 | C_4_H_4_N_4_O_2_ | Uracil | M.H. | 252198525 |
|  | 1.55 | [M+H]^+^ | 245.0766 | 245.0769 | -704 | C_9_H_12_N_2_O_6_ | Uridine | M.H. | 52158984 |
|  | 1.62 | [M+H]^+^ | 182.0811 | 182.0812 | -0.632 | C_9_H_11_NO_3_ | L-Tyrosine | M.H. | 666645366 |
|  | 1.70 | [M-H]^-^ | 117.0179 | 117.0181 | -2.746 | C_4_H_6_O_4_ | Succinic acid | HL/HQ/HB | 384519671 |
|  | 1.75 | [M+H]^+^ | 180.1381 | 180.1381 | -1.272 | C_11_H_18_NO | Candicine | HB/HL | 11372787298 |
|  | 1.96 | [M+H]^+^ | 132.1019 | 132.102 | 0.178 | C_6_H_13_NO_2_ | L-Leucine | M.H. | 866952278 |
|  | 2.01 | [M-H]- | 169.0134 | 169.0131 | 1.54 | C_7_H_6_O_5_ | Gallic acid | M.H. | 42928559 |
|  | 2.06 | [M+H]^+^ | 268.1038 | 268.1037 | -1.01 | C_10_H_13_N_5_O_4_ | Adenosine | M.H. | 2331002890 |
|  | 2.13 | [M+H]^+^ | 132.1019 | 132.102 | 0.293 | C_6_H_13_NO_2_ | L-Isoleucine | M.H. | 1355791053 |
|  | 2.20 | [M+H]^+^ | 252.1089 | 252.1091 | -0.777 | C_10_H_13_N_5_O_3_ | 2'-Deoxyadenosine | M.H. | 241765686 |
|  | 2.23 | [M+H]^+^ | 284.0986 | 284.0988 | -1.206 | C_10_H_13_N_5_O_5_ | Guanosine | M.H. | 979036924 |
|  | 2.23 | [M+H]^+^ | 152.0566 | 152.0567 | -0.502 | C_5_H_5_N_5_O | Guanine | M.H. | 2359862335 |
|  | 2.32 | [M-H]^-^ | 335.0988 | 335.0973 | 4.616 | C_13_H_20_O_10_ | 3-O-(3-Hydroxy, 3-methyl) glutaryl quinic acid | ZZ | 144353305 |
|  | 3.20 | [M-H]^-^ | 335.0988 | 335.0973 | 4.616 | C_13_H_20_O_10_ | 4-O-(3-Hydroxy, 3-methyl) glutaryl quinic acid | ZZ | 133204724 |
|  | 3.41 | [M-H]^-^ | 335.0987 | 335.0973 | 4.318 | C_13_H_20_O_10_ | 5-O-(3-Hydroxy, 3-methyl) glutaryl quinic acid | ZZ | 177702187 |
|  | 3.79 | [M+H]^+^ | 166.0862 | 166.0863 | -0.443 | C_9_H_11_NO_2_ | L-Phenylalanine | HB/HQ/ZZ | 2079564533 |
|  | 4.23 | [M-H]^-^ | 153.0183 | 153.0182 | 0.4927 | C_7_H_6_O_4_ | Protocatechuic acid | M.H. | 207227126 |
|  | 5.66 | [M-H]^-^ | 373.1142 | 373.1129 | 3.502 | C_16_H_22_O_10_ | Gardoside | ZZ | 87899029 |
|  | 5.78 | [M+H]^+^ | 220.1179 | 220.1179 | -0.36 | C_9_H_17_NO_5_ | D-Pantothenic acid | M.H. | 230300835 |
|  | 5.85 | [M+H]^+^ | 190.0861 | 190.0863 | -0.868 | C_11_H_11_NO_2_ | 4-Methoxy-N-methyl-2-quinolone | HB | 137240465 |
|  | 6.37 | [M-H]- | 335.0907 | 335.0914 | -2.089 | C_20_H_16_O_5_ | Glabrone | GC | 4275716 |
|  | 6.51 | [M-H]^-^ | 137.0231 | 137.0233 | -1.485 | C_7_H_6_O_3_ | Protocatechuic aldehyde | HB/HL/ZZ | 118547344 |
|  | 6.53 | [M-H]^-^ | 353.0864 | 353.0867 | -0.874 | C_16_H_18_O_9_ | Neochlorogenic acid | M.H. | 1609039415 |
|  | 6.81 | [M+H]^+^ | 205.097 | 205.0972 | -0.826 | C_11_H_12_N_2_O_2_ | L-Tryptophan | M.H. | 2935605330 |
|  | 6.96 | [M-H]^-^ | 373.1144 | 373.1129 | 3.904 | C_16_H_22_O_10_ | Geniposidic acid | ZZ | 255552411 |
|  | 7.21 | [M-H]^-^ | 391.1251 | 391.1235 | 4.096 | C_16_H_24_O_11_ | Shanziside | ZZ | 701331166 |
|  | 7.28 | [M-H]- | 285.0617 | 285.0605 | 4.266 | C_12_H_14_O_8_ | Uralenneoside | GC | 125777481 |
|  | 7.47 | [M+Na]^+^ | 427.1207 | 427.1211 | -0.826 | C_17_H_24_O_11_ | 6-alpha-Hydroxygeniposide | ZZ | 16697120 |
|  | 7.48 | [M-H]- | 165.0547 | 165.0546 | 0.4049 | C_9_H_10_O_3_ | Phloretic acid | GC | 11474368135 |
|  | 8.10 | [M+H]^+^ | 272.1278 | 272.1281 | -1.139 | C_16_H_17_NO_3_ | Higenamine | HL/HB | 272224116 |
|  | 8.38 | [M+Na]^+^ | 427.1209 | 427.1211 | -0.474 | C_17_H_24_O_11_ | Gardenoside | ZZ | 31766981 |
|  | 8.50 | [M-H]^-^ | 345.0557 | 345.1544 | 3.696 | C_16_H_26_O_8_ | Picroric acid | ZZ | 2472892721 |
|  | 8.85 | [M-H]- | 167.0339 | 167.0339 | -0.091 | C_8_H_8_O_4_ | Vanillic acid | M.H. | 100646560 |
|  | 8.95 | [M-H]^-^ | 375.1299 | 375.1286 | 3.563 | C_16_H_24_O_10_ | Mussaenosidic acid | ZZ | 17896572 |
|  | 9.15 | [M+H]^+^ | 347.1698 | 347.17 | -0.617 | C_16_H_26_O_8_ | Jasminoside G | ZZ | 55287101 |
|  | 9.47 | [M+Na]^+^ | 427.1208 | 427.1211 | -0.615 | C_17_H_24_O_11_ | Paederia hendersonii methyl ester | ZZ | 5965406 |
|  | 9.60 | [M-H]^-^ | 353.0865 | 353.0867 | -0.619 | C_16_H_18_O_9_ | Chlorogenic acid | M.H. | 3584114078 |
|  | 9.64 | [M+H]^+^ | 303.0496 | 303.0499 | -1.02 | C_15_H_10_O_7_ | Herbacetin or 3,5,7,2,6-Pentahydroxyflavanone | HQ | 79876854 |
|  | 9.69 | [M-H]^-^ | 179.0341 | 179.0339 | 0.976 | C_9_H_8_O_4_ | Caffeic acid | HB/HQ/ZZ | 133169201 |
|  | 10.09 | [M+H]^+^ | 347.1697 | 347.17 | -0.963 | C_16_H_26_O_8_ | Jasminoside B | ZZ | 172567343 |
|  | 10.11 | [M-H]- | 193.0494 | 193.0495 | -0.597 | C_10_H_10_O_4_ | Ferulic acid | HB/HL/ZZ | 584597633 |
|  | 10.58 | [M-H]^-^ | 353.0865 | 353.0867 | -0.704 | C_16_H_18_O_9_ | Cryptochlorogenic acid | M.H. | 191172277 |
|  | 10.7 | [M-H]- | 289.072 | 289.0707 | 4.689 | C_15_H_14_O_6_ | Epicatechin | M.H. | 1171658 |
|  | 11.49 | [M+H]^+^ | 342.1696 | 342.17 | -1.241 | C_20_H_23_NO_4_ | Phellodendrine | HB | 50232127516 |
|  | 12.29 | [M-H]^-^ | 549.1827 | 549.1814 | 2.446 | C_23_H_34_O_15_ | Genipin-1-β-D-gentiobioside | ZZ | 313107125 |
|  | 12.51 | [M+H]^+^ | 328.1541 | 328.1543 | -0.776 | C_19_H_21_NO_4_ | Boldine | HL | 348795446 |
|  | 12.55 | [M+H]^+^ | 303.0497 | 303.0499 | -0.723 | C_15_H_10_O_7_ | Quercetin | M.H. | 780007681 |
|  | 12.88 | [M-H]^-^ | 163.039 | 163.039 | 0.189 | C_9_H_8_O_3_ | Cis-4-coumaric acid | M.H. | 37921914 |
|  | 13.29 | [M+H]+ | 397.1854 | 397.1857 | -0.842 | C_20_H_28_O_8_ | Lobetyolin | JG | 2068233 |
|  | 13.44 | [M-H]^-^ | 387.1298 | 387.1286 | 3.143 | C_17_H_24_O_10_ | Geniposide | ZZ | 571722626 |
|  | 13.46 | [M+H]^+^ | 121.065 | 121.0648 | 1.365 | C_8_H_8_O | Acetophenone | ZZ | 3476975784 |
|  | 13.49 | [M+H]^+^ | 227.0912 | 227.0914 | -0.925 | C_11_H_14_O_5_ | Genipin | ZZ | 1525027070 |
|  | 15.16 | [M+H]^+^ | 331.1749 | 331.1751 | -0.784 | C_16_H_26_O_7_ | Jasminoside A | ZZ | 232055405 |
|  | 15.57 | [M-H]^-^ | 681.241 | 681.2389 | 3.022 | C_32_H_42_O_16_ | Pinoresinol diglucoside | HL | 40023461 |
|  | 15.59 | [M+H]^+^ | 331.1749 | 331.1751 | -0.784 | C_16_H_26_O_7_ | Jasminoside E | ZZ | 657253740 |
|  | 15.75 | [M-H]^-^ | 183.1018 | 183.1016 | 1.038 | C_10_H_16_O_3_ | Jasminodiol | ZZ | 755271349 |
|  | 15.85 | [M-H]- | 417.1193 | 417.118 | 3.144 | C_21_H_22_O_9_ | Neoliquiritin | GC | 1669350550 |
|  | 15.92 | [M-H]^-^ | 385.1142 | 385.1129 | 3.315 | C_17_H_22_O_10_ | Mustard acyl glucoside | HB | 5385381447 |
|  | 15.99 | [M-H]- | 463.0887 | 463.0871 | 3.472 | C_21_H_20_O_12_ | hyperoside | M.H. | 780257141 |
|  | 16.18 | [M]^+^ | 322.107 | 322.1074 | -1.116 | C_19_H_16_NO_4_^+^ | Berberrubine | HL | 22249755948 |
|  | 16.66 | [M-H]- | 417.1194 | 417.118 | 3.216 | C_21_H_22_O_9_ | Liquiritin | GC | 14290071262 |
|  | 16.7 | [M-H]- | 549.1617 | 549.1603 | 2.682 | C_26_H_30_O_13_ | Liquiritin apioside | GC | 1445715 |
|  | 16.95 | [M+H]^+^ | 287.0548 | 287.055 | -0.747 | C_15_H_10_O_6_ | kaempferol | M.H. | 452543824 |
|  | 16.95 | [M]^+^ | 324.1228 | 324.123 | -0.631 | C_19_H_18_NO_4_^+^ | Demethyleneberberine | HB/HL | 4802424905 |
|  | 17.11 | [M-H]- | 593.1518 | 593.1501 | 2.855 | C_27_H_30_O_15_ | Vicenin-Ii | GC | 20739998 |
|  | 17.27 | [M]^+^ | 320.0914 | 320.0917 | -1.14 | C_19_H_14_NO_4_^+^ | Coptisine | HL | 50088355217 |
|  | 17.39 | [M-H]- | 549.1616 | 549.1603 | 2.463 | C_26_H_30_O_13_ | Naringenin 7-O-(2-β-D-Apiofuranosyl)-β-D-glucopyranoside | HQ | 10078550154 |
|  | 17.86 | [M]^+^ | 336.1227 | 336.123 | -1.053 | C_20_H_18_NO_4_^+^ | Berbine | HB/HL | 92433978565 |
|  | 17.92 | [M-H]^-^ | 547.1461 | 547.1446 | 2.747 | C_26_H_28_O_13_ | Chrysin-6-C-Pen-8-C-Hex | HQ | 756621653 |
|  | 18.20 | [M-H]^-^ | 547.1459 | 547.1446 | 2.29 | C_26_H_28_O_13_ | Chrysin-6-C-α-L-Arabinoside-8-C-β-D-glucoside | HQ | 7025576941 |
|  | 18.34 | [M-H]^-^ | 581.2244 | 581.2229 | 0.801 | C_28_H_38_O_13_ | (-)-Lyoniresinol-3α-O-β-glucoside | HB/HL | 363766327 |
|  | 18.71 | [M+H]^+^ | 338.1383 | 338.1387 | -1.121 | C_20_H_19_NO_4_ | Jatrorrhizine | HB/HL | 75148902077 |
|  | 18.76 | [M-H]- | 515.1195 | 515.1184 | 2.15 | C_25_H_24_O_12_ | isochlorogenic acid A | M.H. | 42585703 |
|  | 18.87 | [M-H]^-^ | 519.1876 | 519.1861 | 2.951 | C_26_H_32_O_11_ | Pinoresinol glucoside | M.H. | 69717391 |
|  | 18.89 | [M+H]^+^ | 303.0497 | 303.0499 | -0.921 | C_15_H_10_O_7_ | Herbacetin or 3,5,7,2,6-Pentahydroxyflavanone | HQ | 354594747 |
|  | 18.90 | [M-H]^-^ | 609.1467 | 609.145 | 2.822 | C_27_H_30_O_16_ | Rutin | ZZ | 482936185 |
|  | 19.04 | [M-H]- | 623.1988 | 623.197 | 2.797 | C_29_H_36_O_15_ | Isoaceteoside | GC | 803232569 |
|  | 19.08 | [M-H]^-^ | 623.1988 | 623.197 | 2.797 | C_29_H_36_O_15_ | Verbascoside | HQ | 803232569 |
|  | 19.18 | [M-H]^-^ | 547.1459 | 547.1446 | 2.418 | C_26_H_28_O_13_ | Chrysin-6-C-β -D-glucoside-8-C-β -L-arabinoside | HQ | 1893523600 |
|  | 19.22 | [M-H]- | 433.1141 | 433.1129 | 2.809 | C_21_H_22_O_10_ | Chalconaringenin 4-​O-​glucoside | GC | 1528498912 |
|  | 19.52 | [M+H]^+^ | 334.1071 | 334.1074 | -0.792 | C_20_H_15_NO_4_ | Worenine | HL | 762598221 |
|  | 19.57 | [M-H]- | 577.1569 | 577.1552 | 2.908 | C_27_H_30_O_14_ | Isoviolanthin | GC | 682979451 |
|  | 19.66 | [M+H]+ | 579.1705 | 579.1708 | -0.625 | C_27_H_30_O_14_ | Violanthin | GC | 1482564667 |
|  | 19.82 | [M-H]^-^ | 547.1459 | 547.1459 | 2.29 | C_26_H_28_O_13_ | Chrysin-6-C-Hex-8-C-Pen | HQ | 6324512109 |
|  | 19.91 | [M+H]+ | 235.1326 | 235.1329 | -1.237 | C_14_H_18_O_3_ | Lobetyol | JG | 31028036 |
|  | 19.99 | [M-H]^-^ | 579.1725 | 579.1708 | 2.863 | C_27_H_32_O_14_ | 6'-O-trans-sinapoyl gardenoside | ZZ | 350498962 |
|  | 20.01 | [M+H]^+^ | 431.227 | 431.2276 | -1.32 | C_21_H_34_O_9_ | (1R,7R,8S,10R) -7,8,11-Trihydroxyguai-4-en-3-one-8-β-D-glucopyranside | ZZ | 73519355 |
|  | 20.16 | [M-H]- | 301.0718 | 301.0707 | 3.705 | C_16_H_14_O_6_ | Tetrahydroxymethoxychalcone | GC | 486132102 |
|  | 20.16 | [M-H]^-^ | 581.188 | 581.1865 | 2.698 | C_27_H_34_O_14_ | 2 ,4 ,6-Trihydroxydihydrochalcone-3-C-β-D-glucoside-6-O-β-D-glucoside | HQ | 478810016 |
|  | 20.20 | [M-H]^-^ | 345.0617 | 345.0605 | 3.612 | C_17_H_14_O_8_ | Viscidulin III | HQ | 640498487 |
|  | 20.25 | [M]^+^ | 352.154 | 352.1543 | -1.061 | C_21_H_22_NO_4_^+^ | Palmatine | HB/HL | 1.69173E+11 |
|  | 20.59 | [M-H]^-^ | 593.152 | 593.1501 | 3.175 | C_27_H_30_O_15_ | Kaempferol-3-O-glucoside-3''-rhamnoside | ZZ | 177733706 |
|  | 20.73 | [M-H]^-^ | 547.1462 | 547.1459 | 2.637 | C_26_H_28_O_13_ | Chrysin 6-C-β-D-glucoside | HQ | 1587009670 |
|  | 20.91 | [M-H]^-^ | 547.146 | 547.1459 | 2.527 | C_26_H_28_O_13_ | 8-C-α-L-arabinoside | HQ | 2405034024 |
|  | 20.98 | [M-H]^-^ | 579.1723 | 579.1708 | 2.535 | C_27_H_32_O_14_ | 6'-O-trans-sinapoyl gardenoside isomer | ZZ | 228187854 |
|  | 21.26 | [M-H]- | 515.1198 | 515.1184 | 2.732 | C_25_H_24_O_12_ | isochlorogenic acid C | M.H. | 133291815 |
|  | 21.44 | [M-H]^-^ | 187.0967 | 187.0965 | 1.283 | C_9_H_16_O_4_ | Azelaic acid | M.H. | 617276055 |
|  | 21.54 | [M-H]- | 255.0662 | 255.0652 | 3.939 | C_15_H_12_O_4_ | Liquiritigenin | GC | 1592757851 |
|  | 21.83 | [M-H]^-^ | 621.1469 | 621.145 | 3.057 | C_28_H_30_O_16_ | Wogonin-7-O-glu-glu acid | HQ | 312804364 |
|  | 21.97 | [M-H]- | 417.1193 | 417.118 | 3.072 | C_21_H_22_O_9_ | Isoliquiritin | GC | 2975738263 |
|  | 22.04 | [M-H]- | 549.1616 | 549.1603 | 2.354 | C_26_H_30_O_13_ | Licuraside | GC | 2216753327 |
|  | 22.04 | [M-H]- | 549.1616 | 549.1603 | 2.463 | C_26_H_30_O_13_ | Isoliquiritin apioside | GC | 2216753327 |
|  | 22.25 | [M+FA-H]- | 475.1247 | 475.1246 | 2.53 | C_22_H_22_O_9_ | Ononin | GC | 1797495401 |
|  | 22.3 | [M+H]+ | 397.1851 | 397.1857 | -1.597 | C_20_H_28_O_8_ | Platetyolin B | JG | 7196880 |
|  | 22.46 | [M-H]^-^ | 695.22 | 695.2182 | 2.667 | C_32_H_40_O_17_ | 6"-O-trans-p-coumaroyl genipin gentian disaccharide glycoside | ZZ | 1635392189 |
|  | 22.53 | [M-H]^-^ | 475.0884 | 475.0871 | 2.689 | C_22_H_20_O_12_ | 5,7,2'-Trihydroxy-6-methoxy flavone-7-O-glucuronide | HQ | 1015846552 |
|  | 22.6 | [M-H]- | 285.0768 | 285.0757 | 3.824 | C_16_H_14_O_5_ | licochalcone B | GC | 359243197 |
|  | 22.60 | [M-H]^-^ | 755.2411 | 755.2393 | 2.363 | C_34_H_44_O_19_ | 6"-O-trans-sinapoyl genipin gentian disaccharide glycoside | ZZ | 264508718 |
|  | 22.67 | [M-H]- | 285.0771 | 285.0757 | 4.595 | C_16_H_14_O_5_ | Homobutein | GC | 359243197 |
|  | 22.80 | [M-H]^-^ | 725.2308 | 725.2287 | 2.798 | C_33_H_42_O_18_ | 6"-O-trans-feruloyl genipin gentian disaccharide glycoside | ZZ | 103546682 |
|  | 23.12 | [M-H]^-^ | 551.2136 | 551.2123 | 2.281 | C_27_H_36_O_12_ | Sinapoyljasminoside L | ZZ | 280603653 |
|  | 23.24 | [M+H]^+^ | 287.0547 | 287.055 | -1.096 | C_15_H_10_O_6_ | Luteolin | M.H. | 1365889762 |
|  | 23.26 | [M-H]^-^ | 269.0457 | 269.0444 | 4.461 | C_15_H_10_O_5_ | Norwogonin | HQ | 1376907443 |
|  | 23.36 | [M-H]^-^ | 559.146 | 559.1446 | 2.473 | C_27_H_28_O_13_ | 4-O-sinapoyl-5-O-caffeoyl quinic acid | ZZ | 93008651 |
|  | 23.42 | [M+H]+ | 433.1126 | 433.1129 | -0.654 | C_21_H_20_O_10_ | Apigetin-7-O-glucoside | JG | 1973178214 |
|  | 23.81 | [M+H]+ | 255.0649 | 255.0652 | -1.001 | C_15_H_10_O_4_ | Daidzein | GC | 436431452 |
|  | 23.88 | [M+H]+ | 563.1757 | 563.1759 | -0.35 | C_27_H_30_O_13_ | Glycyroside | GC | 117217124 |
|  | 24.01 | [M-H]- | 695.1991 | 695.2006 | 2.954 | C_35_ H_36_ O_15_ | Licorice-glycoside B/D1/D2 | GC | 429062280 |
|  | 24.15 | [M-H]^-^ | 593.1881 | 593.1865 | 2.745 | C_28_H_34_O_14_ | 6'-O-trans-sinapoyl genipin glycoside | ZZ | 293279513 |
|  | 24.54 | [M-H]^-^ | 505.0994 | 505.0977 | 3.372 | C_23_H_22_O_13_ | Viscidulin II 2'-O-β-D-glucuronide | HQ | 927458549 |
|  | 24.56 | [M+Na]^+^ | 485.199 | 485.1993 | -0.604 | C_21_H_34_O_11_ | Jasminoside T | ZZ | 50131300 |
|  | 24.75 | [M-H]^-^ | 445.0777 | 445.0765 | 2.606 | C_21_H_18_O_11_ | Baicalin | HQ | 5662612054 |
|  | 25.29 | [M-H]^-^ | 975.3727 | 975.3704 | 2.36 | C_44_H_64_O_24_ | Crocin I | ZZ | 197861522 |
|  | 25.43 | [M-H]^-^ | 429.0827 | 429.0816 | 2.445 | C_21_H_18_O_10_ | Chrysin-7-O-β-D-glucuronide | HQ | 2979617680 |
|  | 25.52 | [M+H]^+^ | 471.2011 | 471.2013 | -0.431 | C_26_H_30_O_8_ | Limonin |  | 2610000890 |
|  | 25.60 | [M-H]^-^ | 475.0883 | 475.0871 | 2.43 | C_22_H_20_O_12_ | 5,7,8-Trihydroxy-6-methoxy flavone-7-O-glucuronide | HQ | 3498196295 |
|  | 25.67 | [M-H]^-^ | 445.1143 | 445.1129 | 3.067 | C_22_H_22_O_10_ | Wogonin-5-O-β-D-glucoside | HQ | 609979465 |
|  | 25.95 | [M-H]- | 267.0665 | 267.0652 | 4.923 | C_16_H_12_O_4_ | Formononetin | GC | 379115583 |
|  | 26.06 | [M-H]^-^ | 459.0936 | 459.0922 | 3.004 | C_22_H_20_O_11_ | Oroxylin-A-7-O-glucuronide | HQ | 3507424575 |
|  | 26.13 | [M-H]^-^ | 445.0778 | 445.0765 | 2.881 | C_21_H_18_O_11_ | Norwogonin-7-O-β-D-glucuronide | HQ | 2730190826 |
|  | 26.2 | [M-H]- | 269.0821 | 269.0808 | 4.551 | C_16_H_14_O_4_ | Echinatin | GC | 83065118 |
|  | 26.25 | [M+H]^+^ | 593.1864 | 593.1865 | -0.136 | C_28_H_32_O_14_ | Acaciin | HL | 17098163 |
|  | 26.38 | [M-H]^-^ | 475.0883 | 475.0871 | 2.559 | C_22_H_20_O_12_ | 5,6,7-Trihydroxy-8-methoxyflavone-9-O-glucuronopyranoside | HQ | 5660036753 |
|  | 26.48 | [M-H]- | 725.21 | 725.2111 | -1.519 | C_36_ H_38_ O_16_ | Licorice-glycoside A/C1/C2 | GC | 42328730 |
|  | 26.52 | [M-H]^-^ | 461.1097 | 461.1078 | 3.996 | C_22_H_22_O_11_ | (2S)-5,7-Dihydroxy-6-methoxyflavanone-7-O-β-D-glucuronide | HQ | 186246175 |
|  | 26.57 | [M+H]^+^ | 221.0708 | 221.0709 | -0.42 | C_14_H_8_N_2_O | 6H-Indolo(3,2,1-de)(1,5)naphthyridin-6-one | HB | 646155430 |
|  | 26.69 | [M-H]^-^ | 459.0938 | 459.0922 | 3.469 | C_22_H_20_O_11_ | Wogonoside | HQ | 10617349138 |
|  | 26.97 | [M-H]- | 828.4484 | 828.4502 | -2.206 | C_42_H_68_O_16_ | 3-O-β-D-laminaribiosyl polygalacic acid | JG | 47943110 |
|  | 26.97 | [M-H]- | 827.4449 | 827.4459 | 3.055 | C_42_H_68_O_16_ | Platycosaponin A | JG | 103700186 |
|  | 27.24 | [M+Na]^+^ | 837.3143 | 837.3152 | -0.978 | C_38_H_54_O_19_ | Crocin II | ZZ | 16506602 |
|  | 27.31 | [M+H]^+^ | 230.0811 | 230.0812 | -0.301 | C_13_H_11_NO_3_ | γ-Piperine | HB | 225295059 |
|  | 27.40 | [M-H]^-^ | 445.0779 | 445.0765 | 3.018 | C_21_H_18_O_11_ | Norwogonin-8-O-β-D-glucuronide | HQ | 2604472361 |
|  | 27.78 | [M+H]^+^ | 537.2329 | 537.233 | -0.239 | C_27_H_36_O_11_ | 6'-O-trans-Sinapoyljasminoside A | ZZ | 238550911 |
|  | 28.19 | [M-H]^-^ | 489.1042 | 489.1028 | 2.933 | C_23_H_22_O_12_ | 5,7-Dihydroxy-8,2'-dimethoxyflavone-7-O-β-D-glucuronide | HQ | 2465040315 |
|  | 28.21 | [M+H]^+^ | 271.0962 | 271.0965 | -1.197 | C_16_H_14_O_4_ | Alpinetin | HL | 110656824 |
|  | 28.21 | [M+H]^+^ | 535.2171 | 535.2174 | -0.835 | C_27_H_34_O_11_ | 6'-O-trans-Sinapoyljasminoside C | ZZ | 113517774 |
|  | 28.27 | [M-H]- | 255.0662 | 255.0652 | 3.821 | C_15_H_12_O_4_ | Isoliquiritigenin | GC | 458234316 |
|  | 28.46 | [M+H]+ | 845.4518 | 845.4529 | -1.286 | C_42_H_68_O_17_ | Platycoside L | JG | 170584628 |
|  | 28.59 | [M-H]- | 299.0562 | 299.055 | 3.797 | C_16_H_12_O_6_ | kaempferol 3-O-methyl ether | GC | 763753716 |
|  | 29.28 | [M-H]^-^ | 269.0456 | 269.0444 | 4.363 | C_15_H_10_O_5_ | Baicalein | HQ | 5033230887 |
|  | 30.5 | [M-H]- | 837.3923 | 837.3938 | 2.321 | C_42_H_62_O_17_ | Licorice saponin P2 | GC | 875870022 |
|  | 30.59 | [M+H]+ | 679.3683 | 679.3688 | -0.762 | C_36_H_54_O_12_ | platycoside M-1 | JG | 74468323 |
|  | 31.28 | [M+H]+ | 667.4045 | 667.4052 | -0.972 | C_36_H_58_O_11_ | 3-O-β-D-glucopyranosyl polygalacic acid | JG | 167073699 |
|  | 31.28 | [M+H]+ | 799.4465 | 799.4474 | -1.223 | C_41_H_66_O_15_ | Platycodon B | JG | 116275066 |
|  | 31.32 | [M+H]+ | 961.4994 | 961.5003 | -0.916 | C_47_H_76_O_20_ | Platycoside F | JG | 87747521 |
|  | 31.32 | [M+H]+ | 1093.5405 | 1093.5402 | -1.829 | C_52_H_84_O_24_ | Deapioplatycodin D | JG | 186076902 |
|  | 31.46 | [M+H]^+^ | 455.206 | 455.2064 | -1.025 | C_26_H_30_O_7_ | Obacunone | HB/HL | 486945131 |
|  | 31.67 | [M+H]+ | 1001.4578 | 1001.4588 | -0.756 | C_54_H_84_O_26_ | Platyconic acid D | JG | 655294778 |
|  | 31.78 | [M+H]+ | 1077.5469 | 1077.5476 | -0.682 | C_52_H_84_O_23_ | Platycoside J | JG | 17296078 |
|  | 32.00 | [M+H]^+^ | 285.0754 | 285.0758 | -1.228 | C_16_H_12_O_5_ | Oroxylin A | JG | 6120112762 |
|  | 32.03 | [M+H]+ | 1107.5203 | 1107.5194 | -1.385 | C_52_H_82_O_25_ | Platyconic acid C | JG | 101224798 |
|  | 32.07 | [M+H]+ | 517.3158 | 517.316 | -0.348 | C_30_H_44_O_7_ | Platycogenic acid A lactone | JG | 1060691039 |
|  | 32.1 | [M+H]+ | 517.3157 | 517.316 | -0.484 | C_30_H_44_O_7_ | Platycogenic acid B lactone | JG | 1060691039 |
|  | 32.19 | [M-H]- | 819.382 | 819.3833 | 2.683 | C_42_H_60_O_16_ | licorice-saponin E2 | GC | 502602592 |
|  | 32.25 | [M+H]+ | 1135.5521 | 1135.5531 | -0.858 | C_54_H_86_O_25_ | Platycoside C | JG | 110673864 |
|  | 32.28 | [M+H]+ | 1135.552 | 1135.5531 | -0.964 | C_54_H_86_O_25_ | platycoside B | JG | 110673864 |
|  | 32.43 | [M+H]^+^ | 315.0859 | 315.0863 | -1.294 | C_17_H_14_O_6_ | Kumatakenin | HB | 1856684438 |
|  | 33.01 | [M+H]+ | 535.3263 | 535.3265 | -0.476 | C_30_H_46_O_8_ | platycogenic acid A | JG | 93114818 |
|  | 33.01 | [M+H]+ | 845.4531 | 845.4529 | 0.157 | C_42_H_68_O_17_ | PlatycosideK | JG | 31638866 |
|  | 33.17 | [M-H]- | 681.3862 | 681.3845 | 2.607 | C_36_H_58_O_12_ | 3-O-β-D-glucopyranosyl platycodigenin | JG | 65902556 |
|  | 33.6 | [M-H]- | 367.1188 | 367.1176 | 3.228 | C_21_H_20_O_6_ | Glycycoumarin | GC | 127055834 |
|  | 33.6 | [M-H]- | 983.4501 | 983.4517 | 1.866 | C_48_H_72_O_21_ | Licorice saponin A3 | GC | 3487893297 |
|  | 33.94 | [M+H]+ | 535.3262 | 535.3265 | -0.588 | C_30_H_46_O_8_ | Platycogenic acid B | JG | 48865386 |
|  | 34.74 | [M-H]- | 351.0876 | 351.0863 | 3.803 | C_20_H_16_O_6_ | Licoisoflavone B | GC | 327594430 |
|  | 34.81 | [M-H]- | 353.1397 | 353.1384 | 3.935 | C_21_H_22_O_5_ | Gancaonin I | GC | 45533994 |
|  | 35.21 | [M-H]- | 353.1033 | 353.102 | 3.697 | C_20_H_18_O_6_ | Licoisoflavone A or Licoflavonol | GC | 1355015666 |
|  | 35.57 | [M-H]^-^ | 327.1605 | 327.1591 | 4.392 | C_20_H_24_O_4_ | Crocetin | ZZ | 215786826 |
|  | 36.72 | [M-H]- | 337.1446 | 337.1434 | 3.513 | C_21_H_22_O_4_ | Licochalcone A | GC | 1178438 |
|  | 37.55 | [M-H]- | 967.4554 | 967.4568 | 2.191 | C_48_H_72_O_20_ | Rhaoglycyrrhizin | GC | 1108304916 |
|  | 37.93 | [M-H]- | 821.3975 | 821.3989 | 2.493 | C_42_H_62_O_16_ | glycyrrhizic acid | GC | 15655574711 |
|  | 39.20 | [M-H]^-^ | 651.2666 | 651.2647 | 2.868 | C_32_H_44_O_14_ | All-trans-crocetin di-β-D-glucosyl ester | ZZ | 91808107 |
|  | 39.52 | [M-H]^-^ | 651.2667 | 651.2647 | 2.96 | C_32_H_44_O_14_ | Crocin III | ZZ | 211832920 |
|  | 40.85 | [M-H]- | 681.387 | 681.3845 | 3.767 | C_36_H_58_O_12_ | 3''-O-β-D glucopyranosyl platycodigenin | JG | 26073417 |
|  | 40.87 | [M+H]+ | 475.3416 | 475.3418 | -0.339 | C_29_H_46_O_5_ | Platycodonoids A | JG | 14032641 |
|  | 40.9 | [M+H]+ | 637.3942 | 637.3946 | -0.713 | C_35_H_56_O_10_ | Platycodonoids B | JG | 17165366 |
|  | 40.96 | [M-H]^-^ | 487.3434 | 487.3418 | 3.373 | C_30_H_48_O_5_ | Erubigenin | ZZ | 31397626 |
