# Supplementary material for: Uncovering the active constituents and mechanisms of Rujin Jiedu powder for ameliorating LPS-induced acute lung injury using network pharmacology and experimental investigations
Source: Front Pharmacol. 2023 May 11;14:1186699. doi: 10.3389/fphar.2023.1186699 (PMC10210165; doi:10.3389/fphar.2023.1186699)
Supplement: Supplementary file 5 [file DataSheet1.docx]

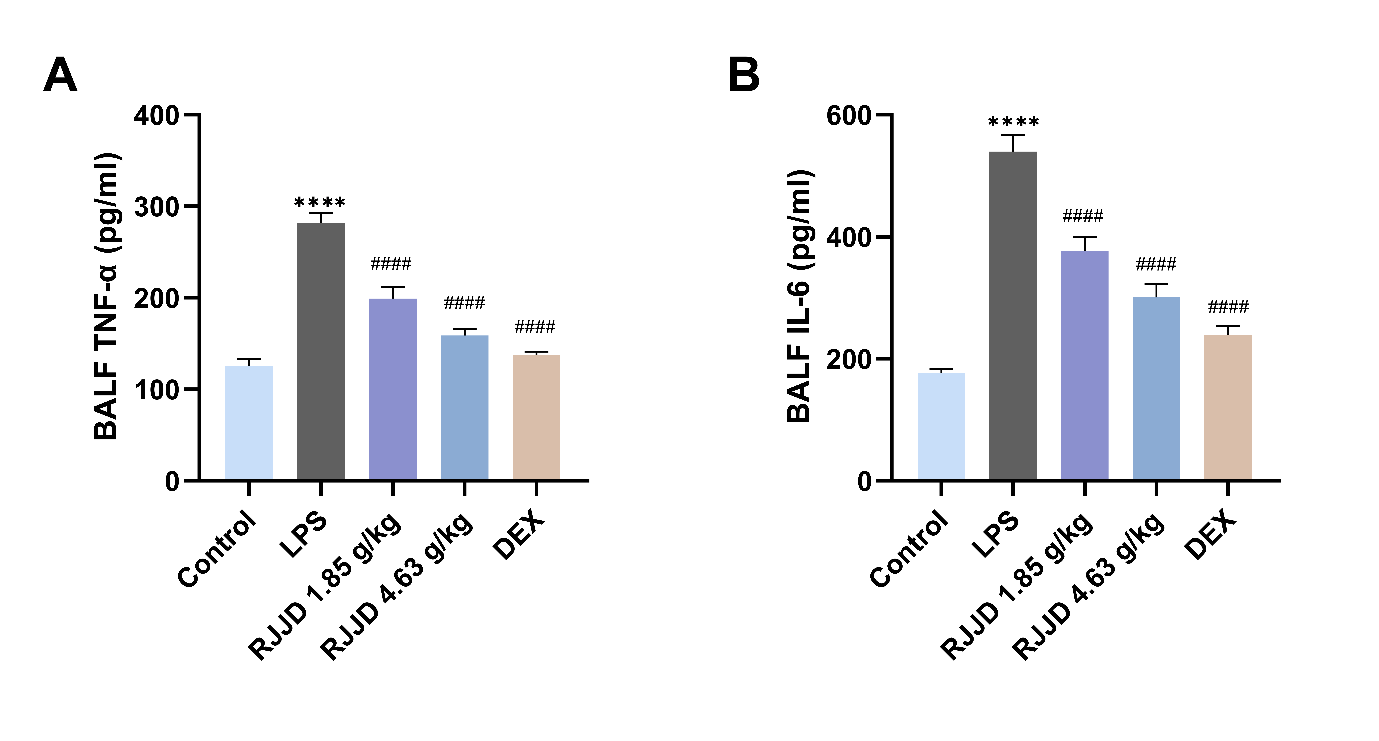


Figure S1 | BALF inflammatory factors (A) TNF-α and (B) IL-6 were detected by ELISA. Data was mean ± SD (n = 5). *****p <* 0.0001 *vs.* Control group; ^####^*p <* 0.0001 *vs.* LPS group.
